# Abstracts from the 24th Congress of the Italian Society of Cystic Fibrosis and the 14th National Congress of Cystic Fibrosis Italian Society: Salerno, Italy. 8 -10 November 2018

**DOI:** 10.1186/s13052-019-0631-0

**Published:** 2019-04-16

## CFTR/CELLULAR STRESS

### A1 Myriocin potential as a phenotype-modifying therapeutical in Cystic Fibrosis

#### Natalia Cirilli^1^, Alessandra Mingione^2,3^, Emeranziana Ottaviano^4^, Fabiola Bonezzi^3^, Michele dei Cas^3,5^, Matteo Barcella^4^, Silvia Tedesco^1^, Tatiana Armeni^6^, Anna Caretti^3^, Rita Paroni^5^, Elisa Borghi^4^, Paola Signorelli^3^

##### ^1^Cystic Fibrosis Care Centre, Mother-Child Department, United Hospitals, Ancona, Italy; ^2^Italian Research Cystic Fibrosis Foundation, Verona, Italy; ^3^Biochemistry and Molecular Biology Laboratory, Health Sciences Department, University of Milan, Milan, Italy; ^4^Microbiology Laboratory, Health Sciences Department, University of Milan, Milan, Italy; ^5^Clinical Biochemistry and Mass Spectrometry Laboratory, Biochemistry and Molecular Biology Laboratory, Health Sciences Department, University of Milan, Milan, Italy; ^6^Dipartimento Scienze Cliniche, Specialistiche- DI.S.C.O., Università Politecnica delle Marche, Ancona, Italy

###### **Correspondence:** Natalia Cirilli (natalia.cirilli@ospedaliriuniti.marche.it)

**Background**

Cystic Fibrosis (CF) is caused by a variety of mutations of the CFTR ion channel, with deletion of phenylalanine 508 (F508del) representing approximately 70% of patients. This mutation induces a proteinopathy, characterized by aggregates of mutated and unfolded proteins, hyper-inflammation, impaired trafficking and altered metabolism at cellular level. CFTR dysfunction has primarily a devastating effect on lungs, causing chronic inflammation and recurrent unresolved infections. CF patients develop severe comorbidities: male infertility, biliary cirrhosis, osteopenia/osteoporosis, renal failure, diabetes (50% of adult patients), malabsorption and increased synthesis of cholesterol and its accumulation in liver but also in other tissues, dyslipidemia with increased plasma triglycerides, pancreas fibrolipomatosis, hepatic lipogenesis and steatosis. Lung chronic inflammation and infection have been extensively associated to the lipotoxin ceramide, core component of membrane lipids. such as sphingomyelins and glycosphingolipids. We previously demonstrated that the sphingolipid synthesis inhibitor Myriocin is able to reduce inflammation and to ameliorate defense response against bacterial and fungal infection.

**Materials and methods**

43 CF patients with selected genotypes were enrolled. Monocytes from peripheral blood samples were extracted and also clinical data were collected.

**Results**

We here demonstrate the mode of action of this molecule. First, we show in IB3 cell line, that Myriocin is an effective inducer of autophagy which is defective in CF proteinopathy and can sustain pathogen clearance (xenophagy). Second, we demonstrated that Myriocin activates key transcriptional factors, TFEB, FOXO1a and PPARgamma involved in autophagy induction, mitochondria genesis and activity, energy production and lipid mobilization and consume. We proved that Myriocin significantly increases the transcription of downstream genes regulating fatty acids entry in mitochondria (CTP1a and 1b; FATP) and their oxidation (ACAD L). Next, we showed that Myriocin drammatically reduces pathological accumulation of lipid un-organized deposits in IB3 cells. By Mass spectrometry we observed that inhibition of sphingolipid synthesis causes a reduced content of non sphingoid-base containing lipids, such as glycerol lipids and cholesterols. We then proved that Myriocin is able to change the transcriptional profile of IB3 treated cells, by gene sequencing analysis, enhancing the transcription of genes involved in lipid transport and consume and energy metabolism that resulted partially downregulated in CF. Finally, Myriocin treatment of peripheral blood monocytes from CF patients, infected with *A. fumigatus* conidia, significantly increases their pathogen killing ability.

**Conclusions**

Myriocin is a potent modifier of pathological gene expression profile in CF and a potential therapy for CF related infection.

### A2 miR-125b/NRF2/CFTR circuitry: a pilot study on oxidative stress in cystic fibrosis

#### Maria Pelullo^1^, Francesca Megiorni^2^, Giuseppe Cimino ^2^, Antonio Pizzuti^3^, Serena Quattrucci^2^, Claudio Talora^1^, Samantha Cialfi^1^

##### ^1^Department of Molecular Medicine, Sapienza University of Rome, Rome, Italy; ^2^Department of Pediatrics, Sapienza University of Rome, Rome, Italy; ^3^Department of Experimental Medicine, Sapienza University of Rome, Rome, Italy

###### **Correspondence:** Samantha Cialfi (samantha.cialfi@uniroma1.it)

**Background**

In physiopathology of Cystic Fibrosis (CF), oxidative stress implications were recognized and widely accepted. The CFTR defects in ionic transport disrupt the intracellular redox balance causing CF classical pathologic hallmarks. Therefore, oxidative stress together with detoxification genes and miRNA aberrant expression could be associated with clinical outcome.

**Materials and methods**

Patients (n=8) with CF diagnosis with the same mutation (F508del), but various clinical status, compared to healthy individuals. After nasal brushing, we extracted total RNA of patients for consecutive expression analysis of CFTR, NRF2 and its targets as well as miR-125b, by quantitative real time PCR (qPCR) using TaqMan chemistry. Written informed consent was obtained by each participant to the study.

**Results**

In this pilot study, we analyzed the existence of a correlation among CFTR, miR-125b, NRF2 and oxidative stress genes in a representative number of CF patients. In CF patients with chronic *P. aeruginosa* (PA) lung infection, the mRNA levels of the NRF2 gene were significantly down-expressed and showed a direct correlation with CFTR gene expression. Interestingly, the NRF2 downmodulation was accompanied by an induced expression of miR-125b. On the contrary, PA negative CF patients showed NRF2 expression levels more similar to those of healthy individuals, with a reduction of miR-125b, even if CFTR levels remained downmodulated. These data are in line with the expression of the oxidative stress target genes NQO1 and GST-T1. Moreover, we found that PA positive patients with a FEV1>60% showed the expression of HMO1, another NRF2 target gene, higher than those with a lower FEV1.

**Conclusions**

The evidence of a CFTR, NRF2 and miR-125b impaired network as oxidative stress response in CF patients, prompt us to hypothesize that these molecular mechanisms could explain the wide CF phenotypic variability as an additional control level over the CFTR gene mutations. Furthermore, this study may allow the discovery of potential therapeutic targets in order to improve CF patient’s quality of life by screening the expression of these oxidative stress factors as prognostic markers.

## MODEL SYSTEMS

### A3 CFTR-Dependent Bicarbonate Transport in Human Rectal Biopsies carryng CFTR variants

#### Paola Melotti^4^, Francesca Rescigno^1^, Alessia Farinazzo^1^, Valeria Esposito^1^, Anna Baruzzi^1^, Stefano Aliberti^2^, Francesco Blasi^3^, Luca Rodella^5^, Francesco Tomba^5^, Filippo Catalano^5^, Angelo Cerofolini^5^, Emily Pintani^4^, Hugo de Jonge^6^, Claudio Sorio^1^

##### ^1^Department of Medicine, University of Verona, Verona, Italy; ^2^Department oh Pathophysiology and Transplantation, Internal Medicine Department, Respiratory Unit and Cystic Fibrosis Adult Center, Fondazione IRCCS Ca’ Granda Ospedale Maggiore Policlinico, University of Milan, Milan, Italy; ^3^Department oh Pathophysiology and Transplantation, Cardio-thoracic Unit and Adult Cystic Fibrosis Center, Fondazione IRCCS Ca’ Granda Ospedale Maggiore Policlinico, University of Milan, Milan, Italy; ^4^Cystic Fibrosis Center, Azienda Ospedaliera Universitaria Integrata, Verona, Verona, Italy; ^5^Endoscopy Unit, Azienda Ospedaliera Universitaria Integrata, Verona, Verona, Italy; ^6^Department of Gastroenterology and Hepatology; Erasmus University Medical Center, Rotterdam, Netherlands

###### **Correspondence:** Paola Melotti (paola.melotti@aovr.veneto.it)

**Background**

Intestinal Current Measurements (ICM) have been utilized for several decades for measuring ex vivo CFTR function as support for difficult diagnosis and for detection of effects of CFTR modulators in preclinical studies, as well as for monitoring therapies of patients affected by cystic fibrosis (CF). Transport of both chloride and bicarbonate is mediated by CFTR, a transmembrane channel permeable to anions. Defective bicarbonate transport plays a role in CF and CFTR related disorders leading to pancreas and lung damage. Selective defect of bicarbonate transport was previously reported for a group of CFTR variants in patients with pancreatitis and no lung disease (LaRusch et al., PLOS Genetics 2014; Park et al., Gastroenterology 2010). We set up ICM in appropriate conditions for detecting selective CFTR mediated/regulated bicarbonate transport defect.

**Materials and methods**

We performed ICM in rectal biopsies of 8 non CF subjects, 8 CF patients and 8 patients with unknown diagnosis referring to the Cystic Fibrosis Center of Verona for CFTR function measurements. ICM were performed according to both the procedure of European Cystic Fibrosis Society ICM Standard Operating Procedure and variations using Cl^-^ free or HCO_3_^-^ free medium and carbonic anhydrase/transport inhibitors in non CF controls vs. wide spectrum of CFTR mutations including CFTR bicarbonate defective mutants.

**Results**

Normal tracings for both anions were observed in non CF controls while abnormal tracings in terms of Cl and bicarbonate transport were obtained in all CF patients except one with Q1463H/R553X/D806G CFTR mutation with pancreatitis with pancreas divisum and lung infection (Staphylococcus aureus methicillin resistant). In subjects with inconclusive diagnosis in the presence of disseminated bronchiectasis we found normal tracings for both bicarbonate and Cl except in two patients with selective defect of bicarbonate transport in the presence of I807M+/-, R668C+/-, CFTR genotypes and Cl sweat values of 18 and 23 mmol/L and 28 and 68mmol/L, respectively (sweat test by Gibson and Cooke performed twice). None had clinical history of pancreatitis in addition to lung disease, evaluation is in progress.

**Conclusions**

Selective defect of bicarbonate transport might be responsible for clinical phenotypes milder than classical CF, including not only pancreas, but also lung disease**.** It can be detected by bioassays such as ICM that is very relevant for understanding functional impact of CFTR variants and their response to CFTR modulators**.** Informed consent was obtained from all subjects.

Study supported by: Italian Cystic Fibrosis Research Foundation grant#7/2016, Lega Italiana FC-Associazione Veneta Onlus.

### A4 Anti-estrogen and direct effects of GB-1877 on calcium-activated chloride channels

#### Roberto Imberti^1^, Lisa Garavaglia^2^, Ivan Verduci^2^, Gaetano Cannavale^2^, Sara Papetti^3^, Giorgio Balduzzi3, Michele Mazzanti^2^

##### ^1^Phase I Clinical Trials Unit and Experimental Therapy, Fondazione IRCCS Policlinico San Matteo, Pavia, Italy; ^2^Department of Biosciences, University of Milan, Milan, Italy; ^3^GB Pharma S.r.l., via Ferreri 11, 27100 Pavia, Italy

###### **Correspondence:** Roberto Imberti (r.imberti@smatteo.pv.it)

**Background**

Although the median age of survival is increased in Cystic Fibrosis (CF) patients, females continue to have a lower median age of survival compared with males. Several lines of evidence indicate that estrogens play a relevant role in the pathophysiology of CF. *In vitro* studies have shown that 17β-estradiol inhibits airway surface liquid production, and *in vivo* studies in CF animals have demonstrated that 17β-estradiol enhances inflammation, increases mucus production and favors the conversion from the non-mucoid to the mucoid form of *P. aeruginosa*. The aim of this study was to investigate the effects of 17β-estradiol and GB-1877 on calcium-dependent chloride channel (CaCC) currents in human bronchial epithelial cells carrying the F508del-CFTR mutation both in homozygosis and in heterozygosis.

**Materials and methods**

Perforated patch clamp experiments were performed on single cells using two immortalized cell lines, CFBE (F508del/F508del) and IB3-1 (F508del/W1282X). Gramicidin (10 or 20 μM) was added to the electrode solution to reach the whole cell configuration. Electrical stimulation protocol consisted in square voltage steps from -80 to +80 mV with 20 mV interval and 800 msec duration.

**Results**

The presence of 17β-estradiol significantly reduced the CaCC currents, both in basal conditions and in the presence of ATP (100 μM). The addition of GB-1877 (10 μM) completely restored the currents abolished by 17β-estradiol, in basal conditions and after stimulation with ATP in both CFBE and IB3-1 cells. GB-1877 had a strong, direct action on membrane current density, which significantly increased more than 4-fold in both cases. The membrane current stimulation produced by GB-1877 was further enhanced by the addition of ATP. CFBE cells incubated for 24 hours with 3 μM VX-809 (a CFTR corrector) and then acutely stimulated with VX-770 (a CFTR potentiator) in the presence of forskolin, showed an increase of chloride currents which were abolished by Inh-172. The chloride current density induced by GB-1877+ATP was, on average, greater than that obtained with VX-809+VX-770+forskolin. The currents elicited by GB-1877+ATP were abolished by the addition of NPPB, a CaCC inhibitor, indicating that the membrane currents recorded in CFBE and IB3-1 cells were mainly carried by chloride through the CaCC. The combined administration of GB-1877/ATP and VXs/FSK had an additional effect on chloride currents.

**Conclusions**

Our results show that GB-1877 restores CaCC currents inhibited by 17ß-estradiol and directly activates the CaCC-dependent chloride currents potentiated by ATP, an effect which is mutation independent. The combined effect of GB-1877 with currently used treatments for CF (e.g., CFTR correctors and potentiators, ENaC inhibitors) could be of benefit to patients with CF.

### A5 Quantification of CRISPR/Cas9 *CFTR* gene editing events using droplet digital PCR

#### Marika Comegna^1^, Francesca Manzoni^1^, Sabrina Maietta^1^, Gustavo Cernera^1^, Federica Zarrilli^2^, Giuseppe Castaldo^1^, Felice Amato^1^

##### ^1^Dept. of Molecular Medicine and Medical Biotechnology, University of Naples Federico II, and CEINGE – Biotecnologie Avanzate, Naples, Italy; ^2^Department of Biosciences and Territory, University of Molise, 86170 Isernia, Italy

###### **Correspondence:** Felice Amato (felice.amato@unina.it)

**Background**

In the last few years, the field of therapeutic gene editing is rapidly developing with the emergence of CRISPR/Cas9 system. This developing technology represents a new opportunity for the treatment of many genetic diseases, including Cystic Fibrosis. However, despite the great potential of this technique, the low frequency of genome editing events, along with the off-target events generation, remains a limitation in the clinical application of this powerful method. In an attempt to develop methods to improve the efficiency of genome editing events, we set up a protocol for quantifying these editing events using the droplet digital PCR (ddPCR) technology.

**Materials and methods**

For this purpose, the ddPCR has already been performed using labeled probe (i.e. fam, hex or vic) technology. Our method, based on EVAGreen technology, uses two primer pairs: one primer pair amplifies a reference sequence distant from the Cas9 cleavage site (reference assay); and a second primer pair include the Cas9 cleavage site (Editing assay). When a reporter expression cassette, included in the HDR construct, is inserted in the Cas9 cleavage site, the amplification of the editing assay is blocked as the amplicon length far exceeds 1000 bp, resulting in a reduction of positive droplets. At this point, comparing the positive droplets from the two assays (reference and editing one) is possible to calculate the percentage of edited allele. The quantification of the genome editing events, using the ddPCR assay, was performed on gDNA of 293T cells transfected with two constructs: 1) one home-made HDR construct (bearing a reporter cassette expressing the puromycin and the yellow fluorescent protein between the homology arm region including the CFTR exon 11, 2) one construct expressing the Cas9 enzyme and the gRNA targeting the genomic sequence of F508del mutation.

**Results**

The combination of the reference and editing assay resulted in a fine quantification of gene editing events. Indeed, we were able to accurately quantify the edited alleles in 293T cells transiently transfected and after puromycin selection.

**Conclusion**

Finally, we demonstrated that the Droplet Digital PCR assay, based on EvaGreen technology, represent an easy and cost effective assay to evaluate the genome editing efficiency, showing in our case, a genome editing efficiency up to 90% of alleles in puromycin selected cells.

We thank Ministero della Salute (Rome, Italy) L.548/93 for the regional research funding quote 2013-2015

### A6 The role of functional studies in the diagnosis and treatment of cystic fibrosis: comparing the case of the G970D and G970R mutation

#### Felice Amato^1^, Paolo Scudieri^2^, Ilaria Musante^2^, Valeria Tomati^3^, Emanuela Caci^3^, MarikaComegna^1^, Sabrina Maietta^1^, Francesca Manzoni^1^, Antonella Miriam di Lullo^1^, Elke De Wachter^4^, Eef Vanderhelst^4^, Vito Terlizzi^5^, Cesare Braggion^5^, Giuseppe Castaldo^1^, Luis J.V. Galietta^2^

##### ^**1**^Dept. of Molecular Medicine and Medical Biotechnology, University of Naples Federico II, and CEINGE – Biotecnologie Avanzate, Naples, Italy; ^**2**^Telethon Institute of Genetics and Medicine (TIGEM), Pozzuoli (NA), Italy; ^**3**^U.O.C. Genetica Medica, Istituto Giannina Gaslini, Genova, Italy; ^**4**^CF Centre, Universitair Ziekenhuis Brussel, VrijeUniversiteit Brussel, Brussels, Belgium; ^**5**^Centro Regionale Toscano Fibrosi Cistica, Azienda Ospedaliero-Universitaria Meyer, Firenze, Italy

###### **Correspondence:** Felice Amato (felice.amato@unina.it)

**Background**

More than 2000 CFTR mutations have been identified so far, but only few of them are clearly defined as CF-causing based on functional studies. We present a case of a rare mutation, the G970D, that has been shown using transfected cDNA in HEK293 cells to be sensitive to ivacaftor. However, a similar missense mutant, G970R in the same codon, was found to be sensitive to potentiators *in vitro* but not *in vivo* due to splicing alteration. Thus, we used several basic research methodologies to evaluate if this patient was eligible for treatment with ivacaftor.

**Materials and Methods**

1) nasal epithelial cells (HNEC) were collected from patients to evaluate the effect of mutations on splicing by RT-PCR assay, 2) HNEC were expanded and polarized for evaluation of CFTR function by ussing chamber system 3) the use of a minigene system was used to confirm *in vitro* the splicing pattern.

**Results**

Firstly, we used *in silico* tool to predict the fisio-pathological effect of mutations, confirming that the G970R completely abolishes the canonical 5’ splice donor site of exon 17 resulting in a likely retention of intron 17. On the contrary, the G970D predicted not to affect splicing. This prediction was confirmed by RT-PCR analysis of mRNA extracted from HNEC cells and from *in vitro* minigene assay. Finally, the functional behavior of CFTR, from HNEC bearing the G970D, was evaluated by short-circuit recordings. The cells responded to cAMP agonist with an increase in trans-epithelial current and this current was nearly doubled by stimulation with ivacaftor. Moreover, in cells that were also incubated with VX-809 (1 μM) for 24 hours, CFTR function was significantly enhanced, with a proportional increase of both cAMP- and potentiator-dependent responses.

**Conclusion**

Our results show that the G970R actually disrupts the RNA splicing thus leading to a severely altered CFTR protein. This event explains the lack of success of treatment with potentiator. In contrast, the G970D does not alter RNA splicing. The resulting G970D mutation affects the gating and trafficking of the CFTR channel that can be targeted with VX-770 and VX-809. These results represent the evidence needed to justify the treatment of the patient with these drugs. Finally, our study is an interesting example of the precision medicine approach by emphasizing the role of appropriate in vitro studies, in this case focused on RNA analysis, to fully characterize the effects of rare CFTR mutations.

We thank Telethon Foundation (TMLGCBX16TT) and Ministero della Salute (Rome, Italy) L.548/93 for the regional research funding quote 2008-2015.

## GENETICS

### A7 Description of Clinical Cases of Disease CFTR Correlated/ Atypical Cystic Fibrosis. Genotype/Phenotype Correlation in Patients with Polymorfism TG12 5T and TG 13 5T

#### Annalisa Ferlisi^1^, Lisa Termini^1^, Elisa Parisi ^4^, Francesca Ficili^1^, Maria A Orlando^1^, Gabriella Traverso^1^, Oriana Bologna^1^, Eleonora Gucciardino^1^, Caterina Di Girgenti^3^, Mariangela Rotolo^4^, Valeria Pavone^4^, Marcella Bertolino^1^, Mirella Collura^1^

##### ^1^UO II Pediatria CRR Fibrosi Cistica e Mal. Respiratorie Osp. Di Cristina- ARNAS CIVICO Palermo, Palermo, Italy; ^2^Patologia Clinica Pediatrica Osp. Di Cristina- ARNAS CIVICO Palermo, Palermo, Italy; ^3^Lab. Genetica Molecolare dell’età evolutiva ARNAS CIVICO Palermo, Palermo, Italy; ^4^Dipartimento di Scienze per la promozione della salute Materno Infantile G. D’Alessandro, Università degli studi di Palermo, Palermo, Italy

###### **Correspondence:** Francesca Ficili (fficili@hotmail.com)

**Background**

Cystic Fibrosis (CF) is the most common genetic disease with an incidence of 1 case per 2500-3000 births. More than 1800 mutations are responsible of a heterogeneous symptoms cortege in relation to the number of organs and systems involved. The classical form is characterized by multi-organ involvement like the exocrine glands. Outside the classical forms are those in which the manifestations of the disease mainly affect a single organ and are generically indicated as “atypical”. In comparison to classical genotypes for CF there are polymorphic variants that if in association with classical mutations are those considered responsible for atypical phenotypes. TG12-13-5T represent polymorphisms whose isolated presence is not relevant in a subject's genotype. Its association with classical mutations responsible for CF, leads to reduced production of cystic fibrosis transmembrane conductance regulator protein (CFTR).

**Cases report**

We describe the cases of five adult, male patients who came to our observation for genetic counseling for infertility. All patients are carriers of the TG12-13 5T polymorphism associated with classical mutations responsible for CF. Mean age was 34.6 years. All patients underwent functional and instrumental respiratory evaluation, pancreatic and hepatic function, sweat testing, ENT evaluation, genetic study for CF. In the cases described, the presence of the TG12 13-5T polymorphism associated with classical mutation responsible for CF correlates with phenotypic pictures characterized by infertility, normal respiratory function, with FEV 1 medium 109% and isolated bronchiectasis, pancreatic and hepatic function were normal, minimal alteration of the liver structure, average pathological sweat test with an average chloride concentration equal to 64.8 mEq/l, only one patient with polyposis and pansinusitis.

**Conclusion**

Although our patients fall under the atypical forms of CF, they have presented multi-organ involvement. Moreover, while the literature data show in these patients variable chloride concentration values, sometimes negative/border line, the average of our case studies showed clearly pathological values.

Informed consent was obtained from all patients for data publication.

### A8 Genotype and phenotype in a patient with R31C and TG12-5T

#### Mirella Collura^1^, Lisa Termini^1^ , Elisa Parisi^3^ , Annalisa Ferlisi^1^, Eleonora Gucciardino^1^, Gabriella Traverso^1^, Oriana Bologna^1^, Caterina Di Girgenti^2^, Tiziana Pensabene^3^ , Francesca Ficili^1^

##### ^1^UO II Pediatria CRR Fibrosi Cistica e Mal. Respiratorie Osp. Di Cristina- ARNAS CIVICO Palermo, Palermo, Italy; ^2^Lab. Genetica Molecolare dell’età evolutiva ARNAS CIVICO Palermo, Palermo, Italy; ^3^Dipartimento di Scienze per la promozione della salute materno infantile G. D’Alessandro. Università degli Studi di Palermo, Palermo, Italy

###### **Correspondence:** Francesca Ficili (fficili@hotmail.com)

**Background**

Cystic fibrosis (CF) is the most common genetically determined, life-limiting disorder in populations of European ancestry. The genetic basis of CF is well established to be mutations in the cystic fibrosis transmembrane conductance regulator (CFTR) gene.CF is a multisystem disease affecting organs where CFTR is expressed (airways, gastrointestinal and reproductive tract).

According to the literature data, variant R31C does not cause CF when combined with another CF-causing variant. Most individuals with this variant (combined with another CF-causing variant) will be healthy. A small number of individuals may develop mild symptoms or be diagnosed with a CFTR-related disorder (CFTR-RD), but symptoms are not expected to be severe enough to meet the definition of CF. There are two scenarios in which making a diagnosis after a positive NBS is less easy:

- borderline sweat test (30-60 mmol/L) without gene mutation;
- normal sweat test with two mutations, at least one of which is of uncertain significance.

There has been a recent Delphi consensus process upon which our management is based and the terminology agreed of ‘CF Screen-positive, Inconclusive Diagnosis (CFSPID).

**Case report**

G.S., male, 5 years. No familiarity with genetic diseases. Full term born, meconium issued in the first 24 hours of life. Positive screening for CF. I level of genetic test was negative for the 32 mutations examined, but presence of polymorphic TG125T site, considered “mild mutation”. He performed several sweat tests in stable conditions with a doubtful or inconclusive result. Sequencing of the CFTR gene showed the presence in exon 2 of the R31C mutation in heterozygosis. The patient had a good staturo-weight growth, normal pancreatic function, mild respiratory symptoms; radio-diagnostics tests show minimal lung lesions. Culture of the sputum shows first Staphylococcus aureus, then Pseudomonas aeruginosa, treated with antibiotic therapy until eradication, and respiratory therapy.

**Conclusion**

The R31C mutation, according to the literature data, is defined as “Non CF causing”, therefore without giving symptoms of disease if not associated with “serious” mutations. Our case is a variant R31C associated with a “mild” mutation (TG12-5T) that presents a clinical suggestive of CF. Can we exclude therefore that this mutation, even associated with non-serious mutations, cannot develop a CFTR-RD picture with mild symptoms or, in males, a possible sterility secondary to agenesis of deferent ducts? What happens in children diagnosed as CFSPID? Many will remain asymptomatic, 10-20% instead will develop symptoms suggestive of CF ( for example PA infection), but the trend is still under study.

Parents gave consent to patient data publication.

### A9 A New CF Causing Insertion in the (TG)Mtn Tract of CFTR Gene

#### Marco Lucarelli^1,2^, Silvia Pierandrei^1,3^, Giovanna Blaconà^1^, Giuseppe Cimino^4^, Benedetta Fabrizzi^5^, Nicole Caporelli^5^, Natalia Cirilli^5^, Marco Cipolli^5^

##### ^1^Dept. of Cellular Biotechnologies and Hematology, Sapienza University of Rome, Rome, Italy; ^2^Italian Pasteur Institute, Cenci Bolognetti Foundation, Sapienza University of Rome, Rome, Italy; ^3^Dept. of Pediatrics, Sapienza University of Rome, Rome, Italy; ^4^Cystic Fibrosis Referral Care Center, AOU Policlinico Umberto I, Rome, Rome, Italy; ^5^Cystic Fibrosis Referral Care Center, Mother-Child Department, United Hospitals, Ancona, Italy

###### **Correspondence:** Marco Lucarelli (marco.lucarelli@uniroma1.it)

**Background**

We present two pediatric clinical cases with diagnosis of Cystic Fibrosis (CF) based on the presence of one mutated allele, F508del, and clinical symptoms. The first case, a female born in 2001 (patient 1), was diagnosed for symptoms at the age of 11 months. The sweat tests were positive (80 and 92 mmol/L) and she presented pancreatic insufficiency, pseudo-Bartter syndrome (loss of salts) at 1 years old and portal hypertension with esophageal varices. Her last forced expiratory volume in 1 second (FEV1) resulted to be 108% of predicted. She presents chronic colonization by *S. aureus* and intermittent by *P. aeruginosa*. The second case, a male born in 2012 (patient 2), was diagnosed by newborn screening with positive value of immunoreactive trypsinogen (IRT, 69 ng/mL), the detection of F508del mutation and positive sweat tests (95 and 102 mmol/L). He presented pancreatic insufficiency and two episodes of distal intestinal obstruction syndrome. His last FEV1 resulted 96% of predicted. Also the patient 2 presents chronic colonization by *S. aureus* and intermittent by *P. aeruginosa*. To complete the genotypes of both patients, an extensive genetic and functional analysis of Cystic Fibrosis Transmembrane conductance Regulator (CFTR) was performed.

**Materials and methods**

DNA samples were extracted from peripheral venous blood and analyzed by DNA sequencing with ABI3130*xl* Genetic Analyzer. RNA samples, obtained from nasal brushing of patients, were extracted, reverse transcribed and amplified by RT-PCR. Results were analyzed by a semiquantitative densitometric assay. The DNA and RNA amplicons were extracted from agarose and sequenced.

**Results**

In both patients, the genetic characterization confirmed the presence of F508del mutation. The analysis of the exon 9 and surrounding intronic regions of CFTR showed an extra amplicon larger (of about 300 nucleotides) than the wild-type. The DNA sequence revealed, in both patients, the presence of an insertion of part of intron 9 in intron 8 of the CFTR gene, within the (TG)m repeat, with a poly-T stretch. The molecular lesion resulted to be the same in both patients, with only a small difference in the number of T in the poly-T stretch. The functional characterization at RNA level revealed a near complete anomalous splicing, without exon 9, from the mutated allele of both patients. Consequently, the mutated allele is expected not to contribute to the formation of functional CFTR protein.

**Conclusions**

This alteration has not been previously described in the literature. Its molecular and functional features are compatible with the definition of new CF-causing mutation of the CFTR gene. This allowed the completion of the genetic characterization of both patients. The fact that the only difference in the two mutated alleles is the small change in the number of T within the poly-T stretch allows speculating about the molecular mechanism of divergence from a common ancestral allele.

We declare to have the informed consent statement for the processing and use of personal data.

### A10 Understanding the network of the regulation of PON2 expression

#### Teresa Carusone^1^, Giovanna Cardiero^2^, Giuseppina Lacerra^2^, Giuseppe Manco^1^

##### ^1^Institute of Protein Biochemistry, National Research Council, Naples, Italy; ^2^Institute of Genetics and Biophysics “A. Buzzati Traverso” National Research Council, Naples, Italy

###### **Correspondence:** Teresa Carusone (t.carusone@ibp.cnr.it)

**Background**

3OxoC12 acylhomoserine lactone (3OxoC12) is the master regulator of Quorum Sensing (QS) regulating the expression of several bacterial virulence. Paraoxonase2 (PON2) has lactonase and anti-oxidant activities that are inhibited by 3OxoC12. PON2, expressed in all tissues, appears involved in many areas of defence of the organisms, including bacterial infections, inflammation, oxidative stress, and innate immune systems. PON2 polymorphic sites (148A/G and 311 S/C) have been previously described to be related to cardiac problems and diabetes type 2. The hydrolysis of 3OxoC12 can be a rational approach to the cure of CF. We focused on the comprehension of the transcriptional and the posttranslational regulation of PON2 expression.

**Materials and methods**

We produced in *E.coli* an engineered version of PON2, with activities matching those described for the native protein, used to generate mutants to understand its structural and biochemical details. We set up qRT-PCRs to highlight the presence of the most abundant mRNA isoforms and to genotype for the two most common PON2 SNP coding variants p.Ala148Gly and p.Ser311Cys. We investigated the regulation of expression of PON2 mRNA, by checking an hypothesis of control via an “mRNA operon”, through the silencing of target genes.

**Results**

We focused to identify the post translational modification and we showed a 3OxoC12-dependent ubiquitination (UBQ) (LYS 168) of PON2 nearby a polymorphic site, able to influence the lactonase activity. Recently a second UBQ site, nearby a second polymorphic in position 311, has been identified by us; then we have produced mutants of the two PON2 polymorphic sites (148A/G and 311 S/C) to study the activities and relationships with PTMs. Studying the mechanism of control of PON2 regulation, we identified a RNA binding protein and a E3 ubiquitin ligase that, when silenced, is able to increase the expression of the PON2 gene. Moreover we genotyped for the SNP 311 several cell lines identifying normal, heterozygotes and also mutant homozygotes.

**Conclusions**

Considering the impact of this gene on the defence against gram-bacterial, its involvement in the inflammation, the indication that the polymorphisms could dictate the severity of disease -as already demonstrated for other pathology- it could be useful to analyze CF patients to understand if the gene can have a role of modifier in relation to the presence of SNPs.

## NEW THERAPIES AND OUTCOME MEASURES

### A11 Cystic fibrosis and new therapeutic strategies: use of Ivacaftor in a pediatric patient

#### Mirella Collura^1^, Annalisa Ferlisi^1^, Lisa Termini^1^, Elisa Parisi^3^, Maria A Orlando^1^, Gabriella Traverso^1^, Oriana Bologna^1^, Caterina Di Girgenti^2^, Valeria Pavone^3^, Marcella Bertolino^1^, Stefania Barrale^1^, Maria A Calamia^1^, Francesca Ficili^1^

##### ^1^UO II Pediatria CRR Fibrosi Cistica e Mal. Respiratorie Osp. Di Cristina- ARNAS CIVICO Palermo, Palermo, Italy; ^2^Lab. Genetica Molecolare dell’età evolutiva ARNAS CIVICO Palermo, Palermo, Italy; ^3^Dipartimento di Scienze per la promozione della salute materno infantile G. D’Alessandro, Università degli Studi di Palermo, Palermo, Italy

###### **Correspondence:** Francesca Ficili (fficili@hotmail.com)

**Background**

Cystic fibrosis (CF) is the most common genetically determined, life-limiting disorder in populations of European. The genetic origin of CF is the mutations in the CF transmembrane conductance regulator (CFTR).CFTR mutations may be classified into 6 different categories based on the mechanisms that are affected. Class I result from mutations leading to absent CFTR production. Class II, including Phe508del, are caused by defective CFTR processing. Class III mutations result in expression of CFTR, the channel gating is defective and results in impaired chloride transport function. Conductance

defects are seen in class IV mutation. Class IV result in a milder phenotype. Finally, class VI mutations are characterized by a functional but unstable CFTR [1]. Ivacaftor is a potentiator that augmented chloride transport and increased airway surface liquid height and cilia beat frequency in airway epithelial cells expressing a CFTR gating mutation (III Class) [2].

**Case report**

C.N. female, 5 years old. Born at the end of gestational age, birth weight 3600 g. Diagnosis by screening and familiarity with FC. Sweat test: 98 mEq/L Cl^-^. Genetics: F508del/G1244E. Intermittent colonization by Pseudomonas aeruginosa and Staphylococcus aureus. Growth curve under 25^0^ centile from the first months of life. She has only 6 months of follow-up. She assumes Ivacaftor at a dose of 75 mg every 12h with good compliance. From the beginning of the therapy program, an improvement of the auxometric parameters and of the score of the CFQ-R respiratory domain was observed; she did not present respiratory exacerbations and showed a good tolerance to the drug, in fact no adverse events were reported. There was not enough compliance to perform the spirometry, necessary for the assessment of respiratory function

**Conclusion**

Kalydeco is an example of innovative therapeutic strategy for carriers of a CFTR channel gating mutation. The further development of such approaches offers great promise for future therapeutic strategies in CF [3].

Parents gave consent to patient data publication.

**References**

1. Sosnay PR, Siklosi KR, Van Goor F et al. Defining the disease liability of variants in the cystic fibrosis transmembrane conductance regulator gene. Nat Genet 2013; 45: 1160–1167

2. Accurso FJ, Rowe S M, Clancy J P, et al. Effect of VX-770 in persons with cystic fibrosis and the G551D-CFTR mutation. N Engl J Med 2010; 363:1991-2003

3. Bell SC, De Boeck K, Amaral MD et al. New pharmacological approaches for cystic fibrosis: Promises, progress, pitfalls. Pharmacol. Ther. 2015;145:19-34

### A12 Therapeutic Benefit of Ivacaftor in Four Cystic Fibrosis Patients Carrying 2789+5g->A CFTR Mutation

#### Matteo Botti^1^, Vito Terlizzi^2^, Paola Catastini^2^, Cesare Braggion^2^

##### ^1^Santa Chiara Hospital, Department of Pediatric, University of Pisa, Pisa, Italy; ^2^Cystic Fibrosis Center, Meyer’s Children Hospital, University of Florence, Florence, Italy

###### **Correspondence:** Matteo Botti (mttbtt88@gmail.com)

**Background**

Ivacaftor (IVA) is a CFTR potentiator approved in Italy in Cystic Fibrosis (CF) patients carrying gating mutations and R117H mutation. In these subjects, IVA was shown to increase percent predicted forced expiratory volume in the 1st second (ppFEV_1_) and BMI and decreased sweat chloride [1,2]. In the last years, the use of IVA is under evaluation for CFTR mutations resulting in residual functioning protein, due to the consistent improve of the chloride transport in vitro and the FEV_1_ in vivo [3,4]. The 2789+5G->A is an alternative splicing mutation of class V associated with reduced synthesis of CFTR protein [5]. We report the use of IVA in four CF adult patients with at least one 2789+5G>A CFTR mutation.

**Materials and methods**

We retrospectively evaluated all CF patients in follow-up at CF Centre of Florence, Italy, carrying at least one 2789+5G->A. We enrolled only patients aged 12 years and older, with a median ppFEV_1_ in the last year ≤ 40% to receive IVA, 150 mg every 12 hours. For each of these, in the year before IVA-starting and on IVA, we evaluated the following characteristics: median ppFEV_1_, best FEV_1_, BMI, Cystic Fibrosis Questionnaire-Revised (CFQ-R) and potential adverse events.

**Results**

Our Centre take care of 26 CF patients heterozygous for 2789+5G>A (female 46.1%, median age: 30.8 years, median ppFEV_1_ in the last year 77 %). We enrolled 4 CF patients (median age: 47 years; in the last year median ppFEV_1_: 29.3%; median best ppFEV_1_: 32.2%; median BMI: 22.5) carrying 2789+5G>A CFTR mutation, and a second CFTR mutation (F508del, W1282X, M1V, 1602delCT). Median IVA exposure was 22 weeks (range: 17-29 weeks). We evaluated clinical picture, lung function and potential adverse events every 8 weeks (range 2-18). During the treatment we observed a mean absolute change of: + 8.3% (range: 4.3 -14.0) for ppFEV_1_, + 7.3% (range: 2-13) for best FEV_1_, + 1.3 (range: 0.4-1.8) for BMI (Table 1). The CFQ-R administered in 3/4 patients before starting IVA and at the last evaluation, showed an improvement of the median respiratory domain scores (+20.6). IVA was well tolerated, no patient reported serious adverse events.

**Conclusions**

Ivacaftor treatment seems to have a potential benefit in CF patients carrying at least one 2789+5G>A CFTR mutation improving lung function, BMI and CFQ-R. Our results, although derived from few patients, support the necessity of further studies to confirm the benefit.

**References**

1. Yu H, Burton B, Huang CJ et al. Ivacaftor potentiation of multiple CFTR channels with gating mutations. J Cyst Fibros 2012; 11:237–45.

2. Moss RB, Flume PA, Elborn JC et al. Efficacy and safety of ivacaftor treatment: randomized trial in subjects with cystic fibrosis who have an R117H-CFTR mutation. Lancet Respir Med. 2015; 3: 524–533.

3. Van Goor F, Yu H, Burton B, et al. Effect of ivacaftor on CFTR forms with missense mutations associated with defects in protein processing or function. J Cyst Fibros. 2014; 13:29-36

4. U.S. National Institutes of Health. ClinicalTrials.gov. Pilot Study Testing the Effect of Ivacaftor on Lung Function in Subjects With Cystic Fibrosis and Residual CFTR Function. Accessed 19-Jun-2017. Last updated: 27-Apr-2018. http://clinicaltrials.gov/ct2/show/NCT01685801.

5. Zielenski J. Genotype and Phenotype in Cystic Fibrosis. Thematic Review Series Respiration. 2000; 67:117-133

**Table 1 (abstract A12).** Results

	duration of IVA-therapy (weeks)	mean data during the year before starting-IVA				mean data during IVA-therapy				mean absolute change			
		ppFEV_1_	best FEV_1_	BMI	CFQ-R	ppFEV_1_	best FEV_1_	BMI	CFQ-R	ppFEV_1_	best FEV_1_	BMI	CFQ-R
patient 1	23	38.8%	43%	25.2	/	43.3%	45%	25.6	/	+4.5	+2	+0.4	/
patient 2	21	39.8%	44%	20.6	83.3	50.0%	52%	22.2	90.2	+10.2	+8	+1.6	+6.9
patient 3	29	11.5%	12%	19.8	50.0	15.8%	18%	21.3	64.6	+4.3	+6	+1.5	+14.6
patient 4	17	27.0%	30%	24.5	33.3	41.0%	43%	26.3	73.5	+14.0	+13	+1.8	+40.2
*median* *(SD)*	22,5	29.3% (13.2)	32.2% (14.9)	22.5 (2.7)	55.5 (25.4)	37.5% (15.0)	39.5% (14.8)	23.9 (2.5)	76.1 (13)	**+8.3** (4.7)	**+7.3** (4.6)	**+1.3** (0.6)	**+20.6** (17.4)

### A13 Long-term effectiveness of ivacaftor in patients with Cystic Fibrosis carrying CFTR mutations with residual function and severe lung disease

#### Donatello Salvatore^1^, Cesare Braggion^2^, Barbara Messore^3^, Giovanna Pisi^4^, Maria A Calderazzo^5^, Michela Francalanci^2^, Carmela Colangelo^1^, Fabiola De Gregorio^1^, Elisa Clivati^3^, Carlotta Biglia^3^, Mimma Caloiero^5^

##### ^1^Cystic Fibrosis Center, AOR Ospedale San Carlo, Potenza, Italy; ^2^ Cystic Fibrosis Center, Hospital Meyer, Florence, Italy; ^3^Adult Cystic Fibrosis Center, Azienda Ospedaliera Universitaria San Luigi Gonzaga, Orbassano, Italy; ^4^Cystic Fibrosis Unit, Department of Pediatrics, University Hospital, Parma, Italyl ^5^Cystic Fibrosis Center, Hospital Giovanni Paolo II, Lamezia Terme, Italy

###### **Correspondence:** Donatello Salvatore (saverdon@gmail.com)

**Background**

Ivacaftor is a CFTR potentiator approved for class-III and *R117H* CFTR mutations and, in the US, for other 28 mutations with “residual function” (RF) of CFTR. RF mutations are usually associated with lung disease that can be delayed in onset and slower in progression, but it may become severe in the adult age.

**Materials and methods**

We describe the effectiveness and safety of Ivacaftor in subjects with CF and severe pneumopathy, with at least one RF mutation. 10 subjects (Male = 1) were included (Median age 48.3 yrs, range 21.7-58.1 yrs). Genotypes were F508del/3849+10KbC-T (n=3), F508del/D579G (n=2), D110H/D1152H (n=1), N1303K/D1152H (n=1), E585X/3272-26A→G (n=1), Delexon22-23/R352Q (n=1), N1303K/R117C. Ivacaftor 150 mg bid was given as compassionate use after approval by the local Ethics Committees. Lung function, use of antibiotics, nutrition, and microbiology were evaluated before starting Ivacaftor and meanly every 3 months in the follow up.

**Results**

Treatment with Ivacaftor resulted in improvement of lung function. The mean absolute change (MAC) in ppFVC and ppFEV_1_ from the baseline value was 7.9 (95% confidence interval [CI], 4.6 to 11.2) and, respectively, 6.5 (95% CI 4.1 to 8.9) after 4 weeks (w). At 24w, the MAC of predicted FVC and FEV_1_ was 16,8% (95% IC 9.4 to 24.2) and, respectively, 12.7 (95% IC 3.44 to 21.9) and at 52w was 12.6% (95% CI 4.6 to 20.5) for FVC and 9.6 (95% IC 3.8 to 15.4) for FEV_1_. Total days of antibiotic therapy dramatically decreased from 480 days (IV 152) in the 6 months before the treatment to 138 days (IV 15) in the 12 months of follow up. No hospitalization was recorded in the follow up. Mean (SD) BMI increased from 23.1 kg/m^2^ (5.8) at baseline to 23.5 (4.3) at 24w and to 25.5 (4.3) at 52w. Sputum microbiology was unchanged. No safety concerns were registered.

**Conclusions**

This case series expand our knowledge about potential benefits of Ivacaftor for CFTR mutations with RF, showing significant and sustained improvement of lung function and BMI and a considerable decrease of pulmonary exacerbations.

### A14 CFTR Modulators effect on physical performance: preliminary data from CF Tuscany casuistry

#### Riccardo Guarise^1^, Beatrice Ferrari^2^, Diletta Innocenti^2^, Sofia Bizzari^2^, Eleonora Masi^2^, Chiara Degl’Innocenti^2^, Michela Francalanci^3^, Cesare Braggion^3^

##### ^1^Tropical and Infectious Disease Department, Careggi, Florence, Italy; ^2^Rehabilitation Unit, Meyer Children Hospital, Florence, Italy; ^3^Cystic Fibrosis Center, Anna Meyer Children's Hospital, Florence, Italy

###### **Correspondence:** Riccardo Guarise (guariser@aou-careggi.toscana.it)

**Background**

CFTR modulators and potentiators opened a new scenario in the treatment of specific genetic defects among CF patients. These new therapeutic options are still undergoing a regulatory and distribution process by Italian healthcare system to become part of drugs portfolio for CF patients. CF Centers throughout Italy were able to prescribe these drugs firstly by a special compassionate use program (CUP) tailored for severely compromised patients and secondly by a regulated program of prescription, administration and monitoring of responsiveness, adverse effects and adherence.

**Materials and methods**

Between July 2014 and September 2018 at the Regional Reference Center for the CF of Florence, a total of 45 patients, 26 females, mean aged 30.4 (6–54) years with CF, carriers of a CFTR channel gating mutation, were enrolled and assigned to receive CFTR modulator therapy with Ivacaftor (Iv) or a combination of Ivacaftor and Lumacaftor (Iv-Lu). For Iv-Lu group, among other medical parameters, spirometry and Six-minute walking test (6MWT) were performed at baseline and after 12-month as functional markers of responsiveness. Data were expressed as mean (range).

**Results**

In our centre to date, 12 patients started Iv, 75% of which commenced it with CUP. Iv-Lu therapy involved 33 patients, 36% of started with CUP. At baseline mean FEV1 % of predicted of Iv group was 61.5 (23-105) and for Iv-Lu group 67.5 (16-161). In both groups the most used airway clearance technique was positive expiratory pressure mask and only 4 patients used periodic-CPAP, both techniques were performed twice a day. Mean period of therapy was 24 months for Iv group and 13 months for Iv-Lu group. By now 15 Iv-Lu patients (45%) completed 12-month of treatment. Among them mean absolute difference of distance walked at 6MWT was -15,2 (+82, -191) meters. 7 patients showed an improvement in 6MWT distance reaching the minimal important difference (45 meters) only in two cases. Also percentage of predicted values of 6MWT distance showed no significant improvement.

**Conclusions**

The high heterogeneity of our casuistry related to respiratory disease and functional capacity underlines the need for a personalized approach to assess the responsiveness to these new drugs. To date data were not available for the entire sample, nevertheless we speculate that a close monitoring of adherence to respiratory physiotherapy should be mandatory and 6MWT distance could be an inaccurate outcome for Iv-Lu responsiveness without the contribution of other more reliable outcomes of maximal exercise performance.

### A15 Effects of Lumacaftor/Ivacaftor on physical activity and exercise tolerance in three adults with Cystic Fibrosis

#### Daniela Savi^1,2^, Stefano Schiavetto^1^, Luigi Graziano^1^, Marco Rivolta^1^, Dario Righelli^3^, Emanuela Leggieri^4^, Giuseppe Cimino^1^, Viviana D’Alu’^1^, Patrizia Troiani^4^, Serenella Bertasi^4^, Paolo Palange^1,5^

##### ^1^Department of Public Health and Infectious Diseases, Adult Cystic Fibrosis Center, Sapienza University of Rome, Rome, Italy; ^2^Cystic Fibrosis Unit, Bambino Gesù Children’s Hospital, Rome, Italy; ^3^Institute for Applied Mathematics “M. Picone” - National Research Council, Naples, Italy; ^4^Department of Pediatrics, Pediatric Cystic Fibrosis Center, Sapienza University of Rome, Rome, Italy; ^5^Eleonora Lorrillard-Spencer Cenci Foundation, Rome, Italy

###### **Correspondence:** Daniela Savi **(**daniela.savi@uniroma1.it)

**Background**

The combination of the cystic fibrosis transmembrane conductance regulator (CFTR) corrector lumacaftor with the potentiator ivacaftor has been approved for the treatment of patients with cystic fibrosis (CF) homozygous for the Phe508del CFTR mutation. The major benefits of lumacaftor–ivacaftor therapy may reside in the reduction of pulmonary exacerbations and in a lower annual loss of lung function. However, there are no reports detailing the effect of lumacaftor–ivacaftor on daily physical activity (PA) and exercise tolerance.

**Materials and methods**

We performed incremental cardiopulmonary exercise testing (CPET) and we assessed PA pre- and post 2 years initiation of lumacaftor–ivacaftor in three adults with CF (CFTR genotype F508del/F508del) and chronic airflow obstruction. CPET-related measurements included oxygen uptake (V’O_2_), carbon dioxide production (V’CO_2_), ventilatory profile, heart rate (HR) and oxygen pulse (V’O_2_/HR) throughout exercise and at lactic threshold (LT) and peak. LT measures represent submaximal exercise related data. PA was assessed using the accelerometer Sense Wear Pro3 Armband.

**Results**

An improvement of daily physical activity following lumacaftor–ivacaftor was accompanied by clinically significant improvements in exercise tolerance**.** Specifically, mild (>3.2 metabolic equivalents (METS)) PA improved by + 13% in patient 1, + 84% in patients 2 and + 89% in patient 3; time spent in PA increased by +59% in patient 2 and +115% in patient 3; active energy expenditure improved + 54% in patient 2 and + 142% in patient 3. V’O_2_ uptake expressed as percentage of predicted significantly increased both at LT and at peak (patient 1 +19 and +33, patient 2 +17 and +42, patient 3 +40 and +20, respectively).

**Conclusions**

Daily physical activity levels and exercise tolerance improved after two years of LUM/IVA therapy, despite no patient education or lifestyle advice.

All patients provided informed written consent for this case report.

### A16 Effectiveness of Ivacaftor in Cystic Fibrosis Patients with Gating Mutations: a Two-Years Single Center Experience

#### Donatello Salvatore, Carmela Colangelo, Fabiola De Gregorio, Giovanni Marsicovetere, Michele D’Andria, Domenica Passarella, Carmela Genovese

##### Centro Regionale Fibrosi Cistica, AOR Ospedale San Carlo, Potenza, Italy

###### **Correspondence:** Donatello Salvatore (saverdon@gmail.com)

**Background**

Ivacaftor is a cystic fibrosis transmembrane conductance regulator (CFTR) potentiator, approved for patients with CF with gating and residual function CFTR mutations. We report the results of a single-center observational study investigating its effects in CF patients with gating mutations.

**Materials and methods**

Patients with gating mutations were recruited to an open-label study evaluating Ivacaftor. Primary outcomes included: lung function, sweat chloride, sputum microbiology, number of pulmonary exacerbations and weight gain.

**Results**

7 subjects (6 females) were enrolled and completed 24 months follow-up on Ivacaftor; mean age at enrollment was 29.3 years (range 12 – 45 years) with 1 subject <18. Two subjects were excluded from the analysis because of very low adherence to the treatment, documented by large variations of sweat Cl and missed collect of Ivacaftor at the pharmacy. Another patient was excluded because not able to perform the lung function tests, having very severe lung disease with Burkholderia cepacia infection. The remaining 4 participants experienced significant improvements in ppFEV_1_ (mean absolute increase of 18% at 1 month that was sustained along the follow up period (24 months); sweat chloride decreased from a mean value of 101 mmol/l at baseline to 39 mmol/L at 24 months. Mean absolute BMI increased of 2.8% after 1 month of treatment until 10.8 % at 12 and 24 months. Pulmonary exacerbations were absent and no subject needed hospitalization during the follow up. Microbiology of sputum was unchanged. No safety concern was reported.

**Conclusions**

Patients with gating mutations experienced improved lung function and nutritional status, and a very pronounced decrease of pulmonary exacerbations. This study supports ongoing use of Ivacaftor for patients with these mutations. Despite the promising nature of Ivacaftor, our data suggest that adherence rates can be suboptimal. Monitoring and motivational interventions are important also for this treatment.

### A17 Effects of Ivacaftor/Lumacaftor in Cystic Fibrosis Patients Homozygous for F508del CFTR Mutation

#### Paola Melotti, Emily Pintani, Gloria Tridello, Ilaria Meneghelli, Marianna Passiu, Elena Spinelli, Sonia Volpi

##### Cystic Fibrosis Center of Verona, Verona, Italy

###### **Correspondence:** Paola Melotti (paola.melotti@aovr.veneto.it)

**Background**

The combination of a CFTR corrector (lumacaftor) and a CFTR potentiator (ivacaftor) is the first CFTR targeted drug approved for clinical use (Orkambi) for patients affected by cystic fibrosis (CF) homoygous for F508del. In placebo-controlled clinical trials (TRAFFIC/TRANSPORT) the mean absolute improvement in the percentage of predicted forced expiratory volume in the 1st sec (FEV1) ranged from 2.6 to 4.0% and the rate of hospitalization or use of intravenous antibiotics was lower in the group treated with Orkambi [1].

**Materials and methods**

We describe the clinical and functional effects on CFTR of this drug in 44 CF patients treated at the CF Center of Verona for at least 3 months: prescriptions and follow up were performed according to the rules of Agenzia Italiana del Farmaco (AIFA). Effects of Orkambi were investigated on nutritional status (weight; Body Mass Index, BMI), CFTR function (sweat chloride by Gibson and Cooke), lung function (FEV1), frequency of pulmonary exacerbations (PE, European Consensus Group definition, Bilton et al., 2011) during the first year of treatment. The duration of therapy ranged from 91 days to 4.8 years.

**Results**

We observed a mean of absolute FEV1 increase of 2.6%,+ 8.4 SD, a mean BMI increase of 0.3, ±1 SD, a mean weight increase of 1.1 kg, ±2.6 SD, a mean reduction of 0.800 +0.941SD of PE requiring IV antibiotics or hospitalization, a decrease of sweat chloride values of 19.2 + 12.5SD vs the mean value of the year before the treatment. In 4 patients the treatment was suspended because of adverse effects, most frequently thoracic oppression. In males and females we obtained very similar results. In severe patients (FEV1 < 50%) we obtained lower improvement of nutritional status than in the others (p<0.05) while other effects were not significantly different in these two groups.

**Conclusions**

CF patients treated with Orkambi at the CF Center of Verona had better nutritional staus, lung function and less PE than before treatment, consistently with their improved CFTR function. Although conditions in clinical use are different than in clinical trials the effects on relevant clinical outcomes have been achieved at levels thereabout expected according to the results obtained in the clinical trials. The variability of response to this drug among homozygous F508del CF patients reported in this study suggest the relevance of personalized medicine and new drug development for CF.

Supported by Lega Italiana FC.

**Reference**

1. Wainwright CE, Elborn JS, Ramsey BW, et al. N Engl J Med 2015; 373:220-231

### A18 Effect of Ivacaftor on comorbidities in a patient with Cystic Fibrosis and pancreatic sufficiency

#### Chiara Cimbalo, Fabiola De Gregorio, Alice Castaldo, Marta Palma, Angela Sepe, Antonella Tosco, Valeria Raia

##### Cystic Fibrosis Center, Pediatric Unit, Department of Translational Medical Sciences University of Naples “Federico II”, Naples, Italy

###### **Correspondence:** Chiara Cimbalo (chiara.cim@outlook.it)

**Background**

Generally patients with Cystic Fibrosis (CF) with pancreatic sufficiency have an increased risk to experience acute recurrent pancreatitis. This occurrence is likely due to diminished ductal flow in pancreatic ducts, with inflammation events and inapt activation of pancreatic digestive enzymes. This risk is related to CFTR dysfunction, causing a lack of fluid secretion. News are emerging on the potential effect of Ivacaftor on comorbidities in CF [1]. We describe a case report about a patient with CF and acute recurrent pancreatitis in treatment with Ivacaftor.

**Case report**

Giada is a 10 years girl, affected by CF (CFTR genotype: G1244E/3849+10kbC>T) with chronic lung disease, bronchiectasis, chronic infection by MRSA, Pseudomonas Aeruginosa intermittent airway colonization and pancreatic sufficiency. From April 2017 she is in treatment with Ivacaftor.

Since 2 years old she had 7 episodes of acute pancreatitis characterized by severe abdominal pain and increase of serum lipase and amylase about 4XULN. All episodes required hospitalization and treatment of symptoms according to standardized protocol. During episodes other causes of acute pancreatitis were ruled-out. Endoscopic retrograde cholangiopancreatography was performed to exclude bilio-pancreatic malformation. Between the acute events serum pancreatic enzymes remained upper normal values.

Already after 8 weeks of Ivacaftor treatment we observed a normalization of both amylase and lipase values that persistently were in the normal range during the 18 months follow-up. The patient has never had pancreatitis. Sweat test decreased from 72 mmol/l to 27 mmol/l. Lung function tests persist in the normal range. Long term follow-up could confirm the effect of Ivacaftor on other comorbidities.

**Conclusion**

Our experience confirms preliminary results about the positive role of Ivacaftor on controlling recurrence of acute pancreatitis in CF. We suggest that the potentiator could ameliorate pancreatic fluid secretion reducing the risk of pancreatic injury.

Patient’s parents gave consent for the publication of clinical data.

**Reference**

Carrion A, Drucy S, Steven D, et al. Reduction of Recurrence Risk of Pancreatitis in Cystic Fibrosis With Ivacaftor: Case Series. J Pediatr Gastroenterol Nutr 2018;66:451-453.

### A19 Real-life effectiveness of Lumacaftor/Ivacaftor in Cystic Fibrosis: a single center experience

#### Donatello Salvatore, Fabiola De Gregorio, Carmela Colangelo, Carmela Genovese, Michele D’Andria, Giovanni Marsicovetere, Domenica Passarella

##### Centro Regionale Fibrosi Cistica, AOR Ospedale San Carlo, Potenza, Italy

###### **Correspondence:** Donatello Salvatore (saverdon@gmail.com)

**Background**

Lumacaftor/Ivacaftor (Orkambi, Vertex Pharmaceuticals) is the first CFTR modulator approved for patients with cystic fibrosis (CF) homozygous for the CFTR F508del mutation. This combination therapy is able to increase the amount of functioning CFTR protein on the cell surface. We report the experience at our center with patients on therapy with Orkambi.

**Material and methods**

We evaluated patients treated with Orkambi since 2016. Patients performed visits after 4 weeks from baseline and then every 3 months. Sweat Cl, BMI, vital signs, physical examination, renal and hepatic profile, spirometry, six-minutes walking test (6MWT), CFQR, sputum culture were evaluated at each visit. All adverse events were registered.

**Results**

17 patients, mean age at baseline 25.4 years (range 15.3-43.2), 3 in the pediatric age (15.9 years, range 15.3-16.9), 6 F, were studied. 17/17 had pancreatic insufficiency, 7/17 CF-related diabetes, 1/17 CF-related liver disease (Child Pugh A), 1/17 was on oxygen therapy. Mean follow-up period was 16.6 months (range 5-24.2). At baseline: mean sweat Cl value 102.5 mEq/l (range 91-111); mean BMI 21 kg/m^2^ (17.5-26.3); mean ppFEV_1_ 67 (range 19-110) and ppMMEF 36 (range 5-111); mean 6MWT 597 mt (range 415-740); mean CFQR respiratory score 67.8 (range 27.7-88.9).Through the follow-up period, mean sweat Cl decreased of 13% since 4 weeks of treatment. No significant variations of BMI, lung function and 6MWT were observed. Pulmonary exacerbations were dramatically reduced in 12/17 patients. Respiratory domain of the CFQ-R score increased with a mean gain of 7 (range 0-22.2) from the baseline, at 1 year. Microbiology was unchanged. Cytolysis and cholestasis lab tests improved in the patient with liver disease. 3/7 patients with diabetes decreased their insulin need. Only 1 patient discontinued treatment after 15 months, because of lung transplant. No safety concerns were reported. Adherence to the treatment was good.

**Conclusions**

The relevant decrease of pulmonary exacerbations rate has been confirmed as the main efficacy measure targeted by Lumacaftor/Ivacaftor. Lung function tests were unchanged during therapy. CFQR-score improvement is probably related to the decrease of pulmonary exacerbation. Use of Lumacaftor/Ivacaftor was safe, even in subjects with severe lung disease and liver disease. Further long term real-life studies, on large number of patients, are needed to evaluate the effectiveness and the prognostic impact of Orkambi.

### A20 Lumacaftor/Ivacaftor: use in a 12-year-old patient with Cystic Fibrosis

#### Mirella Collura^1^, Annalisa Ferlisi^1^, LisaTermini^1^, Elisa Parisi3, Maria A Orlando^1^, Gabriella Traverso^1^, Oriana Bologna^1^, Caterina Di Girgenti^2^, Marcella Bertolino^1^, Sabrina La Fata^1^, Maria R Bonaccorso^1^, Caterina Lo Piparo^1^, Maria A Calamia^1^, Francesca Ficili^1^

##### ^1^UO II Pediatria CRR Fibrosi Cistica e Malattie Respiratorie Ospedale Di Cristina- ARNAS CIVICO Palermo, Palermo, Italy; ^2^Lab. Genetica Molecolare dell’età evolutiva ARNAS CIVICO Palermo, Palermo, Italy; ^3^Dipartimento di Scienze per la promozione della salute materno infantile G. D’Alessandro. Università degli studi di Palermo, Palermo, Italy

###### **Correspondence:** Francesca Ficili (fficili@hotmail.com)

**Background**

Cystic fibrosis (CF) is a life-limiting disease that is caused by defective or deficient cystic fibrosis transmembrane conductance regulator (CFTR) protein activity. Phe508del is the most common *CFTR* mutation. CF affects approximately 70,000 people around the globe including more than 30,000 in Europe. CF is a multisystem disease affecting organs where CFTR is expressed (airways, gastrointestinal and the reproductive tract). Lumacaftor is a CFTR corrector that has been shown to correct Phe508del CFTR misprocessing and increase the amount of cell surface–localized protein. Ivacaftor is CFTR potentiator that increases the open probability of CFTR channels.

**Case report**

M. P., male 12 years old. Diagnosis of CF in complete form with meconium ileus. Sweat test: 96 mEq/L Cl^-^. Genetics: F508del/F508del. Intermittent colonization by Pseudomonas aeruginosa from the first months of life. Poor nutritional situation with slow weight gain. In 2015, diagnosis of GH deficiency, starting from January 2016 GH treatment. In August 2017, in consideration of the “critical need”-status, he started taking the pediatric dosage of Lumacaftor/Ivacaftor as it was part of the compassionate use program. For the first week he took Lumacaftor/Ivacaftor 100/125mg every 12h preceded by short acting bronchodilator. From the second week began full pediatric dosage 200/250mg every 12h. At the age of 12 years of age he continued with the same dosage until the time when the marketing of the association Lumacaftor/Ivacaftor was made available for patients over 12 years of age. During one year of observation the patient presented an improvement of FEV1 equal to 14% of the starting value, and of the FVC equal to 30%; the 6MWT performance showed an increase in the distance traveled of 295 meters measured at the T12. The assessment of the nutritional status of the child showed a slight increase in weight and height, despite the concomitant use of GH treatment, with a consequent increase in BMI of +0.6. During the treatment, no respiratory exacerbations were observed compared to at least 4 episodes that occurred during the previous 12 months. By submitting the patient to the CFQ-R quality of life questionnaire, the score for respiratory symptoms improved significantly from 50 to 83. The sweat test also showed a reduction in chloride concentration. The only parameter that has not undergone changes has been the microbiological data because the patient has maintained colonization by Pseudomonas aeruginosa.

**Conclusion**

The use of this therapeutic strategies gave new prospective of life to the patients who carried a Phe508del mutation in CFTR.

Parents gave consent to patient data publication.

**References**

1. Sosnay, P. R., Siklosi, K. R., Van, G. F. et al. Defining the disease liability of variants in the cystic fibrosis transmembrane conduc- tance regulator gene. Nat Genet 2013; 45: 1160–1167

2. Bell SC, De Boeck K, Amaral MD, et al., New pharmacological approaches for cystic fibrosis: Promises, progress, pitfalls, Pharmacol. Ther. 2015; 145:19-34

3. De Boeck K, Amaral MD. Progress in therapies for cystic fibrosis. Lancet Respir Med 2016; 4 :662-674

### A21 Alternative Therapies in patients with Cystic Fibrosis who carry minimal residual function mutations

#### Antonella Tosco, Fabiola De Gregorio, Paolo Buonpensiero, Alice Castaldo, Chiara Cimbalo, Antonio Di Pasqua, Laura Salvadori, Angela Sepe, Valeria Raia

##### Cystic Fibrosis Center, Pediatric Unit, Department of Translational Medical Sciences University of Naples “Federico II”, Naples, Italy

###### **Correspondence:** Antonella Tosco (antonellatosco@gmail.com)

**Background**

Waiting for all available new modulators could be approved for patients with Cystic Fibrosis (CF) who carry class I and /or class II mutations not homozygous for F508del mutation alternative therapies could be evaluated. A previous phase-2 clinical trial, showed that oral cysteamine (CYS), already approved for the treatment of cystinosis, combined with the kinase-inhibiting epigallocatechin-gallate (EGCG, a safe over-the-counter approved nutraceutical), could be effective in terms of clinical response and functional rescue of CFTR [1]. On the basis of these results we used as off-label therapy this combined treatment for two selected patients.

**Case report 1**

Matteo (genotype: F508del/2183AA>G) is a 7 years boy who presented chronic lung disease, bronchiectasis, liver steatosis, kidney tubular dysfunction and pancreatic insufficiency. One year treatment with CYS (30 mg/Kg qds) plus ECGC (270 mg qd) was completed. During one year follow-up he improved his lung function: FEV1% increased from 92 to 113%, while MMEF% from 64 to 78%. The sweat chloride decreased from 117 mmol/l at baseline to 55 mmol/l after 12 months. No pulmonary exacerbation was registered compared to one exacerbation in the previous year. The patient reports an improvement of daily exercise capacity.

**Case report 2**

Ivana (genotype: N1303K/L1065P) is an 18 years girl affected by a severe lung disease, atelectasis, chronic pansinusitis, liver disease treated with UDCA, frequent respiratory exacerbations requiring hospitalization, multidrug allergy, CF related diabetes and pancreatic insufficiency. She assumed the combined therapy with CYS plus EGCG through 2 years. During two years follow-up she improved lung function: FEV1% increased from 68.7 to 79.9%, while MMEF% from 22.3 to 32.6%. The sweat chloride decreased from 113 mmol/l at baseline to 55 mmol/l after 24 months. The transmembrane conductance flow (measured by SPQ on nasal brushing sample) gained 50% of wild type after 2 years of treatment. Pulmonary exacerbations reduced in severity with a reduction in the number of hospitalizations of 50% (from 6 to 3) during 2 years follow-up.

**Conclusion**

Our experience confirms previous data of efficacy and safety of the association CYS plus EGCG in a selected group of patients. In order to confirm the efficacy of CYS plus EGCG combination in subjects with CF carrying two minimal residual function CFTR mutations, a Phase III randomized-controlled multicentre study has to be encouraged.

Patient’s parents gave consent for the publication of clinical data.

**Reference**

1. Tosco A, De Gregorio F, Esposito S, et al. A novel treatment of cystic fibrosis acting on-target: cysteamine plus epigallocatechin gallate for the autophagy-dependent rescue of class II-mutated CFTR. Cell Death Differ 2017; 24:1305.

## GI/NUTRITION

### A22 Treatment of Distal Intestinal Obstruction Syndrome (DIOS) in cystic fibrosis: proposal of a multicenter protocol

#### Novella Rotolo^1^, Maria Papale^1^, Giuseppe Fabio Parisi^1^, Chiara Franzonello^1^, Annarita Bongiovanni^1^, Lucia Tardino^1^, Mariagrazia Scuderi^2^, Vincenzo Di Benedetto^2^, Salvatore Leonardi^1^

##### ^1^U.O.C. Broncopneumologia Pediatrica e Fibrosi Cistica, AOU Policlinico, Catania, Italy; ^2^U.O.C. Chirurgia Pediatrica, AOU Policlinico, Catania, Italy

###### **Correspondence:** Novella Rotolo (rotolo@policlinico.unict.it)

**Background**

Distal Intestinal Obstruction Syndrome (DIOS) an important morbidity in CF. It is the result of the accumulation of viscid intestinal content combined with sticky mucus occurring usually in the terminal ileum and/or caecum. It may cause a complete obstruction (complete DIOS) or partial obstruction (incomplete DIOS). The most common signs of incomplete DIOS are: progressive cramping abdominal pain, variable distension of bowel sections, palpable mass, occasionally associated with vomiting. The complete DIOS is characterized by biliary vomiting and the plain abdominal radiograph shows the presence of large amounts of feces in virtually all corresponding topography of the small intestine and colonic segments and sometimes dilated bowel loops. Treatment of DIOS is still largely empirical as there are few randomized controlled trials to guide therapy and the last Cochrane on the topic is not clear enough since, currently, there is variation in practice between centers and individual doctors and much of this variation is driven by anecdotal evidence and local experience. Therefore, we propose to begin a multicenter protocol based on literature evidences and our successfully experience, for the treatment of acute episodes of DIOS.

**Materials and methods**

This treatment includes: oral or intravenous rehydration based on clinical conditions; oral administration of N-Acetylcysteine at the dose of 300 mg or 600 mg twice daily; macrogol and sodium or potassium salts at the dose of 40 ml/kg, until desired effect for maximum 1-2 times/die; and finally, i.v. administration of Trimebutin maleate 100 mg every 10 kg up to a maximum of 250 mg diluted in 500 ml of physiological solution. Furthermore, evacuating enemas, such as sodium phosphate bottle 120 ml max for 1 time/day or sorbitol max 1 time/day should be performed if the patient is resistant after 48 hours of a treatment with an osmotic laxative. Enema with Gastrografin can be administered in case of failure of the previous procedure at dosage 20 ml/kg of solution consisting of 100 ml of Gastrografin diluted in 500 ml of physiological solution.

**Results**

Preliminary results are ongoing to evaluate the efficacy of protocol.

**Conclusions**

This protocol has been shown to be useful in CF patients with acute episodes of DIOS and it may be proposed for a multicenter study involving all Italian CF centers.

### A23 Lung clearance index and 24 h pH-metry in Cystic Fibrosis: which link?

#### Giuseppe F Parisi, Maria Papale, Lucia Tardino, Enza Mulè, Novella Rotolo, Salvatore Leonardi

##### UOC Broncopneumologia Pediatrica e Fibrosi Cistica – Università degli Studi di Catania, Catania,, Italy

###### **Correspondence:** Giuseppe F Parisi (giuseppeparisi88@hotmail.it)

**Background**

Gastroesophageal reflux (GER) is common in patients with cystic fibrosis (CF) and is often regarded as playing a role in the pathogenesis of CF lung disease. Individuals with CF have many predisposing factors to the development of GER, with a reported prevalence ranging from 35 to 81%. Both the acid and nonacid components of GER may have an effect on lung disease. The lung clearance index (LCI) is a measure of ventilation distribution obtained from the multiple-breath washout (MBW) test. As the MBW test uses relaxed tidal breathing without the requirement of any maximal effort, it is an attractive test for infants and pre-school-aged children. LCI is abnormal in a significant proportion of pre-school children with cystic fibrosis (CF), and correlates with the presence and extent of structural lung disease detected via chest computed tomography (CT) and lower respiratory tract inflammation and infection.

**Materials and methods**

We enrolled a cohort of 14 patients with CF both children and adults who were investigated with 24-h pH-metry. All patients were free from proton pump inhibitors (PPI) therapy for 1 month and in a stable phase of the disease. At the same time (one day before or after the exam) we performed the study of Lung Clearance Index (LCI) with Multiple Breath Washout (MBW) technique.

**Results**

We found the presence of relevant episodes of acid gastroesophageal reflux in 10 (71%) patients (6 adults and 4 children). Of these patients, all the 6 adults and only 1 child out of 4 had higher LCI values than normal. Among pediatric subjects with normal 24-h pH-metry 2 presented normal values of LCI and 2 abnormal.

**Conclusions**

In general, 71% of subjects present acid gastroesophageal reflux confirming that GER is common in patients with CF. In detail, all adults (100%) seem to show a link between abnormal LCI and 24-h pH-metry. Differently, only half (50%) of children showed abnormal 24-h pH-metry and only 1 of them showed an abnormal LCI. According to our preliminary data, now we are performing 24-h pH-impedance test in children to establish the weight of basic reflux in children with CF. Further studies are needed to validate implications for Lung disease in patient with Gastroesophageal Reflux in CF.

### A24 Intestinal inflammation may correlate with ultrasound abnormalities in Cystic Fibrosis

#### Andrea Catzola^1^, Valentina Angelini^2^, Maria Grazia Caprio^2,3^, Alice Castaldo^1^, Maria Brunella Cipullo^2^, Marta Palma^1^, Antonella Tosco^1^, Angela Sepe^1^, Valeria Raia^1^

##### ^1^Cystic Fibrosis Center, Department of Translational Medical Sciences University of Naples “Federico II”, Naples, Italy; ^2^Department of Advanced Biomedical Sciences, University of Naples Federico II, Naples, Italy; ^3^Institute of Biostructure and Bioimaging, National Council of Research, Naples, Italy

###### **Correspondence:** Andrea Catzola (andreacatzola@gmail.com)

**Background**

Inflammation in patients with Cystic Fibrosis (CF) is not limited to the respiratory tract, but plays an important role also in the gastro-intestinal tract. Fecal calprotectin (FC) is a marker of intestinal inflammation, however its use in CF is controversial. Recent studies support the use of intestinal ultrasound to assess the degree of inflammation in chronic bowel disease.

we have investigated the relationship between high FC levels and the intestinal wall thickening, measured by ultrasound examination, or clinical manifestations in CF patients.

**Materials and methods**

Data collected for the analysis include age, gender, CFTR mutations, sonographically measured colonic wall thickness, FC levels measured by ELISA, pancreatic insufficiency status (defined as fecal elastase value < 200 μg/g stool), FEV1 as a marker of lung function.

**Results**

40 CF patients were consecutively enrolled (22 males; mean age 9.6±4.2 years; range 3-17 years); 33/40 were pancreatic insufficient and 6/40 had also severe liver disease. FC values were over the cut-off in 16/40 patients (normal value <90 ug/g feces), all these patients were pancreatic insufficient. Bowel ultrasound imaging showed an increased wall intestinal thickening in 15/40 patients, an increased appendicular thickness (range 5-6.8 mm) in 8/15, in 4/15 an increased thickness of terminal ileal stricture, in 1/15 patient both structures were involved. We have correlated the FC values with increased wall intestinal thickening, founding the following results: both parameters were abnormal in 6/40 patients; 9/40 had normal FC value with an increased wall intestinal thickening and 10/40 had increased FC value in absence of intestinal abnormalities. Among 7/40 patients with pancreatic sufficiency only 2 showed an increase of intestinal wall. FEV1 was available in 31/40 CF patients and 10/30 showed values between 40% and 80%. Only 3/10 patients showed an increased wall intestinal thickening and nobody had increased FC. Among 6 patients with severe liver disease 3 showed an increased wall thickening of which only one has elevated FC. No correlation was found between pathological wall intestinal thickening and/or FC and clinical data such as recurrent abdominal pain, meconium ileus at diagnosis and distal intestinal obstruction syndrome.

**Conclusions**

Our results show a lower percentage of patients with wall intestinal thickening than data previously reported. In patients with CF, FC could not be a marker of intestinal inflammation as reported in chronic inflammatory bowel disease. Our data show 38% of enrolled patients with elevated FC in disagree with literature [1-2]. Currently preliminary data are not sufficient to reinforce the hypothesis of a correlation between inflammatory marker and wall thickness in children with CF.

**References**

1. Rumman N, Sultan M, Chammas KE, Vi et al. Calprotectin in Cystic Fibrosis. BMC Pediatrics 2014; 14:133

2. Parisi GF, Papale M, Rotolo N, et al. Severe disease in Cystic Fibrosis and fecal calprotectin levels. Immunobiology 2017, 222: 582–586

### A25 Two cases of Cystic Fibrosis onset with acute pancreatitis and a slow resolution

#### Annalisa Ferlisi^1^, Maria G Sciarrabone^2^, Carmela Fondacaro^2^, Maria C Vella^2^, Gabriella Traverso^1^, Lisa Termini^1^, Maria A Orlando^1^, Oriana Bologna^1,^, Sabrina La Fata^1^, Francesca Ficili^1^, Mirella Collura^1^

##### ^1^U.O. Pediatria II per la Fibrosi Cistica (CRR) e le Malattie Respiratorie. Arnas Civico, Palermo, Italy; ^2^Dipartimento di Scienze per la promozione della salute Materno Infantile G. D’Alessandro, Università degli studi di Palermo, Palermo, Italy

###### **Correspondence:** Francesca Ficili (fficili@hotmail.com)

**Case report 1**

Ilary, 7 years, comes to our observation for acute slow-resolution pancreatitis. Second-born from normal course of pregnancy, emission of meconium occurred within the first 24 hours of life. Normal stature-weight growth. During the hospitalization a positive result of the sweat test was carried out and consequently the genetic study was performed with a compound heterozygosis of the CFTR gene (F508del / T338I mutation); the latter is associated with a slightly expressed phenotype, especially with regard to the pancreas secretory function.

**Case report 2**

Giulia, 11 years old, anything relevant in remote medical history, regular weight-weighted growth. She arrives in emergency area for abdominal pain in the epigastric site radiated to the back, with increasing intensity, not responsive to antalgic therapy. At the blood chemistry tests, high values ​​of pancreatic enzymes are found. Excluding infectious and malformative causes of acute pancreatitis, in the suspicion of cystic fibrosis (CF), a sweat test resulted positive , and genetic testing showed mutation in heterozygosity of G542X and 711 + 3A> G intron 5. The latter mutation intervenes on the splicing of CFTR and allows the synthesis of a partially functional protein, or a reduced portion of normally functioning CFTR.

In both cases other exams such as broncho-aspirate culture, chymotrypsin and faecal elastase were in normal and it was undertaken fasting, infusion support and subsequently therapy with pancreatic extracts and ursodeoxycholic acid, with gradual normalization of enzyme levels.

**Conclusion**

Pancreatitis is a relatively rare complication affecting 1-2% of CF patients and may be an initial isolated manifestation of the disease and therefore be the first indicator for the diagnosis of CF. This complication occurs in cases with a functioning pancreas, so in order to manifest inflammation, it is necessary that the organ is still able to secrete enzymes. Approximately 10% of CF patients maintain good pancreatic function until adulthood or for life: they have at least one mutation of the CFTR gene defines as mild. In some of these mild forms, the pancreas causes partial stasis of secretion in the pancreatic canaliculi, inducing inflammation with important abdominal pain and marked increase in pancreatic enzymes in the serum. Follow-up is important for understanding the possible onset of pancreatic insufficiency. In the presence of pancreatitis history, without triggering causes, both in a child and in an adult, it is advisable to investigate for CF.

Parents gave consent to patient data publication.

### A26 Elastography as a diagnostic tool to assess liver disease in Cystic Fibrosis

#### Maria G Caprio^1,2^, Maria B Cipullo^2^, Valentina Angelini^2^, Andrea Catzola^3^, Alice Castaldo^3^, Marta Palma^3^, Antonella Tosco^3^, Angela Sepe^3^, Valeria Raia^3^

##### ^1^Institute of Biostructure and Bioimaging, National Council of Research, Naples, Italy; ^2^Department of Advanced Biomedical Sciences, University of Naples Federico II, Naples, Italy; ^3^Cystic Fibrosis Center, Department of Translational Medical Sciences, University of Naples Federico II, Naples, Italy

###### **Correspondence:** Maria G Caprio (mariagrazia.caprio@ibb.cnr.it)

**Background**

Cystic Fibrosis (CF) is an autosomal recessive genetic disease. CF-associated Liver Disease (CFLD) is a common complication and represents the third cause of death in 2.5% of patients with CF. In clinical practice liver biopsy is not routinely applied to evaluate the severity of liver disease in CF, as it is a painful and invasive procedure. Since much more studies support the use of liver ultrasound to correctly assess the degree of hepatic involvement, we aimed to study a group of children diagnosed as having CF to evaluate the correlation between liver stiffness assessed by ARFI elastograghy and conventional US qualitative examination.

**Material and methods**

Data were collected from 34 consecutively enrolled CF patients including age, gender, antropometric measures, CFTR mutations. Liver stiffness derived from acoustically generated tissue shear wave propagation (Shear-wave elastography-SWE) and Ultra Sound (US) parameters of liver pathology such as hepatomegaly, variation in echogenicity, fibrosis, signs of portal hypertension were also registered in all patients in a bladder manner.

**Results**

Preliminary results include data from 34 CF patients (17 Males; mean age 8.9 years, range 3.3-15.6). All of them had pancreatic insufficiency and carried minimal residual function mutations on both alleles. 10/34 (29.41%) (8 Males; mean age 12.3 range 7.9-15.0) patients showed hepatic stiffness over the cut-off value (range 6.4-15.0) compared to normal value < 6.3 kPa. In 8/10 patients liver stiffness alteration was concordant with US signs of hepatic abnormalities (steatosis, fibrosis), while 2/10 didn’t show any other sign of liver pathology. In 24/34 patients with liver stiffness in the normal range 6 patients showed US qualitative signs of mild liver abnormalities as steatosis and one with fibrosis.

**Conclusions**

According to previous data our preliminary results show a correlation between conventional US and increased values of liver stiffness, examined by Shear-Wave elastograghy [1] in order to detect CFLD. More data are needed to identify liver stiffness as a diagnostic tool of liver disease with advantage of real-time imaging and lower cost and to correlate it to clinical characteristics.

**Reference**

1. Aqul A1, Jonas MM, Harney S, Raza R, et al. Correlation of Transient Elastography With Severity of Cystic Fibrosis-related Liver Disease. J Pediatr Gastroenterol Nutr. 2017; 64: 505-511.

### A27 Transitory Pancreatic Insufficiency in mild form of Cystic Fibrosis: the importance of follow-up with Fecal Elastase-1

#### Claudia Conforti^1^, Piercarlo Poli^2^, Silviana Timpano^2^, Chiara Perrotti^1^, Angela Adreoletti^1^, Elena Gennari^1^, Rita Padoan^2^

##### ^1^Graduate School in Pediatrics, Department of Pediatrics, Children’s Hospital, ASST Spedali Civili Brescia, Brescia, Italy; ^2^Regional Support Center for Cystic Fibrosis, Department of Pediatrics, ASST Spedali Civili Brescia, Brescia, Italy

###### **Correspondence:** Piercarlo Poli (piercarlo.poli@gmail.com)

**Background**

About 85-90% of Cystic Fibrosis (CF) patients has pancreatic insufficiency (PI). PI involves the CF patients with two CFTR “severe” mutations (functional class I, II, III), while the presence of at least one copy of an allele of class IV-V correlates in the majority of cases with a pancreatic sufficiency (PS) phenotype. Although genotype predicts the development of PI there is a clinical variability due to the interaction of other genes (e.g. inflammatory response genes) and environmental factors. The loss of pancreatic function generally appears during the first months and years of life, thus pancreatic function is an important clinical marker of the progression in CF. In order to treat the malabsorption improving the nutritional status it is important to determine the pancreatic functional status since the first month of life in screened babies. In clinical practice the most common pancreatic function test used is measurement of Fecal Elastase-1 (E1) as a good marker of pancreatic status. The test had a 93% sensitivity and a 93% specificity using a <200 ug/g feces cut off. Normally E1 reaches adult levels in the first two weeks of age, thus this method is useful for screening PI/PS in screened infants.

**Cases report**

Here we present 4 cases of CF females carrying at least one “mild” mutation of CFTR gene. In the first months of life, they had E1 insufficient levels (Table 1), then infants were labeled as PI and pancreatic enzyme replacement therapy (PERT) was quickly begun.

Monitoring pancreatic status during clinical follow-up, we assisted to a spontaneous increase of E1 levels, that reached stably PS values allowing us to stop PERT and improve their quality of life.

**Conclusion**

Genotype predicts the maintainance of exocrine PS in CF, as the presence of at least one CFTR mutation of functional class IV-V generally correlate with PS, but there is a clinical variability determining fluctuation of E1 values. We reported four “mild” CF cases initially labeled as PI in the first months of life, however during the follow-up we assisted to a spontaneous raise of E1 values reaching PS range as expected from their genotype. Monitoring regularly pancreatic status also in PI children who carry CFTR mutations with a residual function is necessary to avoid unnecessary PERT.

The families of the patients have given written consent for the publication of the data

**Table 1 (abstract A27).** Clinical characteristics

CFTR GENOTYPE DATE OF BIRTH	FECAL ELASTASE 1 VALUE mcg/g of feces (sampling date)	FECAL FATS (normal value <3%)	AGE AT STOP PERT (year)
F508del/2789+5G>A 22/06/2012	30 (10/7/12)	17%	1
	108 (2/10/12)	12%	
	271 (31/12/12)	-	
	348 (24/06/13)	<3%	
	472 (1/9/14)	-	
	436 (28/9/15)	-	
F508del/2789+5G>A 07/09/2006	<15 (October 2006)	-	8
	169 (April 2007)	-	
	64 (20/12/07)	20-33%	
	495 (27/8/13)	<3%	
	500 (8/10/13)	<3%	
	471 (31/12/13)	5-12%	
	500 (30/04/14)	<3%	
	>500 (28/9/15)	-	
F508del/R1066H 30/05/2014	82,82 (23/06/14)	21-10-10%	4
	>500 (22/02/18)	-	
	>500 (22/03/18)	-	
	>500 (27/04/18)	-	
M1T/M1T 13/12/2010	50-193 (07/03/11)		5
	95 (14/03/14)	31-32% (07/03/11)	
	123 (26/03/13)	<3%	
	147 (31/05/13)	-	
	146 (9/12/13)	<3%	
	208-210 (14/05/15)	<3%	
	492 (17/2/16)	29%	
	307-373 (27/03/17)	4%	
	500-400-216 (22/11/17)	-	

### A28 Percutaneous Endoscopic Gastrostomy (PEG): the Experience of The Regional Reference Center for Cystic Fibrosis of Milan

#### Arianna Giana^1^, Lauretta Valmarana^2^, Rossella Valmarana^2^, Anna MC Bulfamante^1^, Fabiola Corti^1^, Rita M Nobili^1^, Silvia Bellapi^1^, Carla Colombo^1,3^

##### ^1^CF Cystic Fibrosis Regional Center, IRCCS Ca’ Granda Foundation Ospedale Maggiore Policlinico, Milan, Italy; ^2^Department of Clinical and Community Sciences, University of Milan, Milan, Italy; ^3^Department of Medical-Surgical Physiopathology and Transplantation, University of Milan, Milan, Italy

###### **Correspondence:** Arianna Giana (agiana.13@gmail.com)

**Background**

Malnutrition is a complication of Cystic Fibrosis (CF) and has been recognized as a negative prognostic factor. Therefore malnutrition has to be corrected and a step wise approach has been recommended including enteral nutrition (EN) via percutaneously placed gastrostomy (PEG) tube [1]. The aim of this study was to investigate the efficacy of EN via PEG to improve nutritional status, respiratory function and incidence of complications.

**Materials and methods**

A retrospective cohort study on CF patients who underwent PEG between 2000 and 2016 was performed at our Center. Sixteen patients were included in the study with a median age of 13 years, 94% with pancreatic insufficiency. The anthropometric data were recorded 6 months prior PEG placement, at the time of placement and 6 months after. Patients were classified according to CF Foundation (CFF) nutritional criteria. Malnutrition was considered to be present in patients with BMI <10th percentile up to 19 years and <18.5kg/m² over 20 years. In addition, in 15 parents of 13 patients, the level of parental stress was investigated through a self-administered questionnaire; results of PEG-group (n=4) were compared to a group of control subjects with a CF child but not needing EN.

**Results**

Malnutrition was present in almost all patients who underwent PEG, with the exception of 3 children in whom PEG was necessary due to total aversion to oral feeding. After 6 months of EN median values of the Z-score of W/L or BMI increases by 1 standard deviation. At the start of EN, 13 patients (81.3%) were malnourished; 6 months after the malnourished subjects decreased to 31.3% (N=5).

Complications (mechanical, gastroenterological, metabolic) occurred in 50% of patients and were solved; only in one case NE was stopped. The mean percentile value of the Total Stress levels falls within the norm in both groups. Only one mother reported a value in the range of clinical interest due to the coexistence of other problems in addition to CF.

**Conclusions**

Patients needing PEG constitute a small minority in CF population; from our experience it emerges that EN via PEG is safe and helps to achieve an adequate weight when usual interventions fail. Patients with PEG must be evaluated from a nutritional point of view at every clinical check and supported in the management of this device.

**Reference**

1. Smyth AR, Bell SC, Bojcin S, et al. European Cystic Fibrosis Society Standards of Care: Best Practice guidelines. J Cyst Fibros. 2014;13: S23–S42.

### A29 Nutritional status in the first 2 years of life of children with Cystic Fibrosis

#### Anna Coruzzo, Assunta Elia, Giuseppe Scidà, Giuseppina Napoletano, Clara Smaldone, Simona Spadarella, Giada Zollo, Sara Polizzi

##### Cystic Fibrosis Center, Pediatric Unit, Department of Translational Medical Sciences University of Naples “Federico II”, Naples, Italy

###### **Correspondence:** Anna Coruzzo (anna.coruzzo@unina.it)

**Background**

optimizing growth from early childhood in Cystic Fibrosis (CF) is critical, as lung function and survival correlate strongly with nutritional status. Guidelines recommend a persistent optimal nutritional status. The objective of this preliminary study is to evaluate nutritional status in a cohort of children with CF in the first 2 years of life, in follow-up at Cystic Fibrosis Centre of Naples

**Materials and methods**

Data from 12 children (5 Males) affected by CF, born between 2015 and 2016, were retrospectively evaluated. CF was diagnosed in 10/12 by neonatal screening and in 2/10 for suggestive symptoms but with negative newborn neonatal screening. 9/12 were pancreatic insufficient (PI). We evaluated data about nutritional status (body weight, height and P/A ratio with the relative percentile) according to CDC growth curves, feeding modalities, prescriptions of nutritional supplements, clinical outcome and vitamin D levels. Furthermore, we investigated mothers anxiety, evaluated by STAI -Y questionnaire, in relation to nutritional status of enrolled children.

**Results**

Results of anthropometric parameters are summarized in Table 1.

4 patients at a high risk of malnutrition improved through personalized nutritional intervention at 2 years of age. From the analysis of feeding modalities, 4/12 children were breastfed, 5/12 were formula-fed exclusively and 3/12 received mixed feeding. The dietary intake for children was about 120% (range 100%-130%) estimated average requirement. Insufficient children received PERT at a dosage increasing from 2500 lipase/g fat units at the diagnosis to 3300 lipase /g fat units at 24 months of follow-up in order to correct malabsorption. In particular, steatocrit values normalized on average from 34% (range 12%-40%) to 2.32% ( 1% - 6.5%) All enrolled children received salt supplementation. Despite vitamin supplementation in all children since diagnosis only one with severe pancreatic insufficiency showed hypovitaminosis D.

Beyond their nutritional status preliminary data concerning 8 mothers showed a level of anxiety (trait: 35-63 state: 33-72) measured by STAI -Y questionnaire with no correlation to nutritional status.

**Conclusions**

Early individualized nutritional intervention is useful to ensure adequate growth and correct nutritional status in CF. More data are needed to correlate nutritional status and mother anxiety that could impact on rescue of malnutrition.

**Table 1 (abstract A29).** Results

Lenght pc	At diagnosis N=12	2 years N=12	Weight/lenght pc	At diagnosis N=12	2 years N=12
<5°p	4	0	<5°p	0	0
10°-25°	0	1	10°-25°	2	2
25°-50°	2	3	25°-50°	3	3
50°-75°	6	5	50°-75°	6	6
75°-90°	0	3	75°-90°	1	1

pc=percentile

### A30 Severe anemia as rare onset clinical sign of Cystic Fibrosis

#### Novella Rotolo, Giuseppe Fabio Parisi, Maria Papale, Annarita Bongiovanni, Lucia Tardino, Salvatore Leonardi

##### UOC Broncopneumologia pediatrica e fibrosi cistica – Università degli Studi di Catania, Catania, Italy

###### **Correspondence:** Novella Rotolo (rotolo@policlinico.unict.it)

**Case Report**

We report the case of a male, only one son of unrelated parents, born at the 37th week by caesarean section, weight at birth of 2900 gr. At the age of 3 months, he came to our attention for suspected cystic fibrosis (CF) due to the positivity of neonatal screening. The patient appeared very pale and edematous, but in good general conditions and normal vital parameters. We decided to perform a blood count, the sweat test and directly the genetic screening for CF. The examination of the blood count showed a severe normocytic anemia: Hb 5.5 g/dl RBC 2,000,000/mmc, MCV 87 fL, PLT 364.000/mmc, WBC 10.430/mmc. The child was hospitalized, and he underwent other laboratory tests that showed hypoproteinemia (4.7 g/dL), hypoalbuminemia (1.43 g/dL) and increased hemolysis indices (total bilirubin 4.7 mg/dL, direct bilirubin 1.8 mg / dL). The first clinical suspect was a hemolytic anemia, then excluded. He performed parenteral albumin supplementation and blood transfusion with normalization of laboratory values. The sweat test was pathological and the genetic test for CF identified the presence in heterozygosis of two CFTR variants (c.1521_1523delCTT, ([delta]F508 e CFTR:c.3846G>A (W1282X)) related to CF.

**Conclusion**

In the literature there are currently only two recent works of Sismanlar T. of 2016 and Aricò M. of 2018 describing the onset of CF with severe anemia and hypoproteinemia, as rare symptoms related to poor intestinal absorption. Parents gave consent to data publication.

## INFECTION/ MICROBIOLOGY

### A31 Is Serum Amyloid A a marker for diagnosing cystic fibrosis pulmonary exacerbation?

#### Piercarlo Poli^2^, Michael Colpani^1^, Alice Meneghin^1^, Camilla Dallavilla^1^, Silviana Timpano^2^, Rita Padoan^2^

##### ^1^Graduate School in Pediatrics, Department of Pediatrics, Children’s Hospital, ASST Spedali Civili Brescia, Brescia, Italy; ^2^Regional Support Center for Cystic Fibrosis, Department of Pediatrics, ASST Spedali Civili Brescia, Brescia, Italy

###### **Correspondence:** Piercarlo Poli (piercarlo.poli@gmail.com)

**Backgroung**

Identification of pulmonary exacerbation (PE) in CF patients is important for proper treatment. Serological markers as C-reactive protein (CRP) and leukocyte (WBC) counts are not very specific and not included in the Fuchs criteria for PE diagnosis. Serum amyloid A (SAA) proteins are a family of apolipoproteins expressed or secreted by liver during the acute phase of inflammation. SAA genes are regulated by the proinflammatory cytokines (IL-1, IL-6, TNF-α) and induced rapidly under inflammatory conditions like exposure to bacterial LPS. SAA is also involved in amyloidogenesis and tumor pathogenesis. At low concentrations (10-100ng/ml), as in early inflammation, SAA induces chemokines or matrix degrading enzymes and functions as a chemoattractant. When an infectious or inflammatory stimulus persists, the liver continues to produce SAA that becomes a direct bacterial opsonin or interferes with viral infection of host cells. AA amyloidosis is a rare, but severe complication of CF with predominant renal involvement. Aim of the study is to evaluate the usefulness of SAA as a marker of inflammation and a diagnostic criterion for PE.

**Materials and methods**

Between January 2016 and December 2017 we collected serum SAA, CRP and WBC at the time of admission and at discharge for PE (defined according to the Fuchs criteria) in 22 CF patients. Details of the study population are shown in table 1.

**Results**

A total of 40 hospital admissions for PE, in 22 patients (M/F: 11/11, mean age at admission: 11.5 years, range 1-21) were analysed. All 40 events met the Fuchs criteria for PE. The mean serum SAA at admission was 166.5 ng/ml (range 2-937 ng/ml, normal value: < 6 ng/ml). The mean serum CRP at admission was 21.65 mg/l (range <2.9-139 mg/l, normal value: < 2.9 mg/l).

16/40 (40%) had a negative CRP at admission while only 4/40 (10%) had a negative SAA (p<0.05). 4/16 (25%) events with negative CRP had also a negative SAA. WBC were in the normal range for age in 36/40 events (90%). At the time of discharge the SAA was negative in 28/40 (70%) events (mean SAA: 9.9 ng/ml, range <6-74 ng/ml).

**Conclusions**

Literature is still discussing the definition of PE. New and more sensitive biochemical markers of early inflammation are necessary to diagnose PE. SAA may represent a more sensitive biochemical marker than CRP and WBC for PE diagnosis and might be included, after an adequate validation study, in the definition criteria for PE.

**Table 1 (abstract A31).** Characteristics of the study population

Patients (n°)	22		
Sex (M/F)	11/11		
Pulmonary exacerbation (n°)	40		
Pulmonary exacerbation/patients	1.8		
Pancreatic sufficiency (n°)	1 (4.5%)		
MRSA chronic pulmonary infection (n°)	4 (18%)		
PA chronic pulmonary infection (n°)	13 (59%)		
Other infection (MSSA, H.influenzae)	5 (23%)		
Mean age at admission (years)	11.47 (1-21)		
Mean duration of hospitalization (days)	15 ±2		
	Before therapy	After therapy	P-value
Mean serum SAA (ng/ml) (range)	166.5 (2-937)	9.9 (<6-74)	<0.05
Mean serum CRP (mg/l) (range)	21.65 (<2.9-139)	4.7 (<2.9-18)	<0.05

MRSA: methicillin resistant staphylococcus aureus; PA: Pseudomonas aeruginosa; MSSA: methicillin sensitive staphylococcus aureus

### A32 Palivizumab prophylaxis to prevent respiratory syncytial virus infection in infants with Cystic Fibrosis (CF). Experience of the last five years

#### Annalisa Ferlisi^1^, Francesca Ficili^1^, Gabriella Traverso^1^, Lisa Termini^1^, Maria A Orlando^1^, Federico Barooty^2^, Mirella Collura^1^

##### ^1^U.O. Pediatria II per la FIBROSI CISTICA (CRR) e le Malattie Respiratorie, ARNAS Civico, Palermo, Italy^; **2**^Dipartimento di Scienze per la promozione della salute materno infantile G. D’Alessandro, Università degli Studi di Palermo, Palermo, Italy

###### **Correspondence:** Francesca Ficili (fficili@hotmail.com)

**Background**

Respiratory syncytial virus (RSV) is associated with worsening respiratory symptoms in children affected with CF increasing also the hospital admission. Palivizumab is the only preventive therapy, since no vaccine against VRS is now available. No conclusive data are available regarding the use of Palivizumab in CF population because few studies have been carried out.

**Material and methods**

All patients diagnosed with CF and in follow up to our Unit born between 2014 and 2018 were included in a retrospective study. All data on hospital admissions caused by respiratory disease were collected until September 20, 2018. RSV testing was performed from nasopharyngeal aspirates. All patients received Palivizumab in the first year of life.The aim of our study was to obtain epidemiologic data on the incidence of RSV infection in children with CF less than 24 months of life who received Palivizumab.

**Results**

We identified a cohort of 36 patients in follow up in our Unit (F 22, M 14) with CF born between 2014 and 2018 with a diagnosis by screening or symptoms before 24 months of life, who received Palivizumab ( five doses, 15 mg/kg for dose) in the first year of life. 31 of them had a diagnosis for screening and 5 for symptoms. Two patient with CF diagnosed for symptoms are awaiting vaccination in the next October due to a recent diagnosis. Three patients showed a RVS infection during admission in hospital for respiratory symptoms, so the incidence of RSV infection in our cohort is 8,8%. One patient born at 28+3 weeks of gestational age, affected with meconium ileus with ileostomia received two doses of Palivizumab before showing RSV infection. Another patient born full term with meconium ileus treated with barium enema received one dose of Palivizumab before showing RSV infection. The last patient born full term that did not present other risk factors and no other comorbidity, received one dose of Palivizumab before showing RSV infection. All the other patients never presented RSV infection.

**Conclusions**

Although there are no absolute indications on the use of Palivizumab in CF patients, our study showed that two patients with other risk factors have had RSV infections and only one have had RSV infection without other risk factor. It seems to be useful and reasonable to use prophylaxis with Palivizumab in patients with respiratory chronic disease like CF.

### A33 Successful ceftazidime-avibactam treatment of post-surgery *Burkholderia multivorans* genomovar II bacteremia and brain abscesses in a young lung transplanted woman with Cystic Fibrosis (CF)

#### Valeria Daccò^1^, Laura Claut^1^, Luca Castellazzi^1^, Francesca Garbarino^1^, Antonio Teri^2^, Carla Colombo^1^

##### ^1^Cystic Fibrosis Center, IRCCS Ca’ Granda, Ospedale Maggiore Policlinico, University of Milan, Milan, Italy; ^2^Cystic Fibrosis Microbiology Laboratory, IRCCS Cà Granda, Ospedale Maggiore Policlinico, University of Milan, Milan, Italy

###### **Correspondence:** Valeria Daccò (valeria.dacco@policlinico.mi.it)

**Background**

CF patients are vulnerable to many bacterial infections throughout their life. *Burkholderia cepacia* complex (Bcc) pathogens consist of at least 20 closely related species of which *B. cenocepacia* and *B. multivorans* account for approximately 85-97% of all Bcc infections in CF patients. Chronic Bcc infection, especially by *B. Cenocepacia*, is associated with a more rapid decline in lung function and considered a relative or absolute contraindication to lung transplantation in almost all transplant centers for a markedly adverse impact on survival. Bcc infections are particularly difficult to treat due to their intrinsic multidrug resistance to many antibiotics, including aminoglycosides, cephalosporins, polymyxins and traditional antipseudomonal β-lactams. One of the most common resistance mechanism in *Burkholderia* species is the production of β-lactamases. Non beta-lactamase-mediated resistance, like efflux pumps and reduced outer membrane permeability plays also a role in the decreased susceptibility of BCC species towards beta lactam antibiotics. Avibactam is a novel β -lactamase inhibitor that restores the susceptibility to ceftazidime by preventing enzymatic hydrolysis. The combination of ceftazidime/avibactam (CEF-AVI) represents an important new therapeutic option for Bcc, as shows the highest in vitro susceptibility and may be a potential salvage therapy for Bcc infections when the other treatment options have failed.

**Case Report**

A critical CF woman with pancreatic insufficiency, B multivorans chronic airway colonization, CF-related diabetes, end stage renal disease on haemodialysis treatment received bilateral lung transplantation at the age of 32 years. After six months she underwent to FESS procedure for the treatment of syntomatic polypous pansinusitis and subsequently developed multiple brain abscesses and bacteremia due to *B. multivorans* genomovar II resistant to ceftazime, aminoglycosides, levofloxacin, piperacilline/tazobactam and intermediate to meropenem. This severe infection did not improve despite standard multiple antibiotic regimen: meropenem combined with trimethoprin-sulfamethoxazole and levofloxacin. After the introduction of CEF-AVI, a new β -lactam/β-lactamase inhibitor combination, she achieved clinical recovery, radiological and biochemical improvement and was discharged with no subsequent consequences.

**Conclusion**

We report for the first time the efficacy of CEF-AVI in combination with other antibiotics for the treatment of a severe systemic B multivorans infection in a lung transplanted CF patient and end stage renal disease. No adverse events were documented during the treatment carried on for 102 days. Our report highlights one of the possible severe complication that *B. multivorans* may have in an immunosuppressed patient after lung transplantation. CAZ-AVI may be a potential savage therapy for BCC severe infection even if the recommended dose in CF has to be still standardized.

Consent for publication

written informed consent was obtained from the patient.

### A34 Ceftolozane/tazobactam for the treatment of MDR Pseudomonas aeruginosa in a patient suffering for Cystic Fibrosis waiting for lung transplant

#### Mirella Collura^1^, Salvatore Giordano^2^, Gabriella Traverso^1^, Lisa Termini^1^, Francesca Ficili^1^, Annalisa Ferlisi^1^, Oriana Bologna^1^, Chiara Iaria^3^, Elisa Parisi^3^, Tiziana Pensabene^4^, Marcella Bertolino^1^, Maria A Orlando^1^

##### ^1^Pediatric Unit for the Cystic Fibrosis, Allergy and Respiratory Diseases. ISMEP, Palermo, Italy; ^2^Pediatric Infectious and Tropical Diseases Unit. ISMEP, Palermo, Italy; ^3^Department of Science for Health Promotion and Mother and Child Care, University of Palermo, Palermo, Italy; ^4^Microbiologic Unit Arnas Civico Palermo, Palermo, Italy

###### **Correspondence:** Mirella Collura (mirella.collura@arnascivico.it)

**Background**

Cystic Fibrosis (CF) is an autosomal recessive disease due to the mutation of the gene that encodes the Cystic Fibrosis Transmenbrane Regulator (CFTR), which regulates and is involved in the transport of electrolytes. Incidence: 2.000-3.000 new cases a year. CF is a multisystem disease affecting organs where CFTR is expressed (airways, gastrointestinal and the reproductive tract). The main pathogenic germs are *Staphylococcus aureus* (Sa), *Pseudomonas aeruginosa* (Pa). In CF a targeted and aggressive therapy of chronic Pa infections is one of the mainly strategy to allow the improvement of the prognosis. The new combination of a β-lactam and a β-lactamase inhibitor such as Ceftolozane-Tazobactam shows a rapidly bactericidal action, a linear kinetics, it spreads well in the lung and acts on numerous mechanisms of resistance of Pa.

**Case report**

Female aged 16, weighing 51 Kg, suffering from CF in a complete form, waiting for lung transplants, is hospitalized for fever and dyspnoea. EGA and blood exams showed a septic state. She arrived with Levofloxacin, Tobramycin, Meropenem antibiotic iv treatment. Cultural examinations were performed and started antibiotic therapy with Ceftolozane/Tazobactam and Phosphomycin. From the fifth day of therapy the clinical conditions gradually improve and also the blood tests showed a reduction in CRP. Therapy is continued through 20 days. In the meantime, a subsequent sputum culture examination isolated 2 strains of Pseudomonas (mucoid and wrinkled) with a sensitivity similar to that of the entrance except for a resumption of sensitivity to Piperacillin-Tazobactam. So we decided to modify the therapy from ceftalozane to Piperacillin/Tazobactam and Colistina iv. After 7 days the clinical conditions worsened, with a reappearance of fever and increase in inflammation indices. The last sputum culture test documented the reappearance of resistance to Piperacillin-Tazobactam, therefore we decided to stop the therapy and to resume the association Phosphomycin+ Ceftolozano/Tazobactam. There was a progressive improvement of the clinical conditions.

**Conclusion**

The patient came to our observation in a highly compromised general state, that is, with an exacerbation of her chronic Pa MDR infection. We started a combination therapy with the aim of improving the clinical outcome i waiting for the lung transplant. The choice fell on Ceftolozano-Tazobactam due to its strong anti-pseudomonas action, used with good clinical evidence also in rescue therapies. We did not observe any side effects and the hepato-renal and medullary parameters remained in the normal range.

Parents gave authorization to patient data publication.

### A35 Longitudinal metagenomic 16s sequencing of a CF infant’s respiratory samples as a tool for microbiome monitoring

#### Ramona Pezzotta^1^, Piercarlo Poli^2^, Erika Scaltriti^3^, Alberto Fattori^1^, Silviana Timpano^2^, Simona Fiorentini^1^, Rita Padoan^2^

##### ^1^Section of Microbiology, Dept. of Molecular and Translational Medicine University of Brescia/ASST-Spedali Civili of Brescia, Brescia, Italy; ^2^Cystic Fibrosis Regional Support Centre, Children’s Hospital, ASST - Spedali Civili, Brescia, Italy; ^3^IZSLER, Risk Analysis and Genomic Epidemiology Unit, Parma, Italy

###### **Correspondence:** Ramona Pezzotta (ramypezzotta@gmail.com)

**Background**

Pulmonary environment plays a major role in modulating CF lung disease, thus, accurate identification of the whole respiratory microbiome has a crucial role for the management of patients, the improvements of their healthcare and quality of life. CF infants’ microbiome analyses can give a better understanding on the associations between bacterial populations and patient conditions during their lives, starting from the very beginning, and can better show how this relationship affects pulmonary microenvironment and the evolution of CF disease. Sequencing analyses and comparison with cultural approaches were performed on 10 clinical samples that cover the entire life of a single 2-years patient to obtain a longitudinal study on the variation of the respiratory microbiome compared to the culture-based methods.

**Materials and methods**

From January 2015, ten samples of nasopharyngeal aspirates were collected from a single patient starting from the first microbiological exam of his life. These samples firstly underwent to microbial analysis performed by routine cultural standards for CF samples, stored by frozen, and, retrospectively, were processed by New Generation Sequencing (NGS). Briefly bacterial DNA was extracted using Qiagen kit and 16s library preparation protocol was adjusted for poor quality samples (as infants’ ones are). Raw reads taxa were assigned by BLAST and visualized by MEGAN software.

**Results**

NGS demonstrated how commensal flora undergoes to several modifications during sampling period, underlying the presence of different families never discriminated by microbial cultures such as anaerobic population (Prevotellaceae and Fusobacteriaceae) that were quite abundant (up to 18%) into the microbiome. Also, variations of different families were observed over time and prevalence of *Staphylococcus aureus*, considered by culture the main pathogen in this patient, was sometimes different if compared with families identified by NGS.

**Conclusions**

Nowadays, the culture-based methodology is considered the gold standard for microbiological diagnostics: although it has high specificity, this method has low sensibility due to several problems, such as the difficult isolation and detection of pathogens from the polymicrobial flora, the presence of unculturable pathogens and anaerobes that cannot be detected using standard culture-based conditions and the presence of pathogens extremely adapted to the pulmonary microenvironment that grow after several days (up to 7 days) under peculiar conditions (growth at 30°C or in micro-aerobic atmosphere). For all these reasons, culture-based methodology cannot characterize all the different bacterial populations in each sample and so culture independent technologies, such as NGS technology, are needed to overcome the previous issues.

Consent for publication

The patients gave the consent to publish clinical data.

### A36 The role of nasal washes in patients with Cystic Fibrosis affected by chronic rhinosinusitis

#### Serena Buonaurio^1^, Paola Iacotucci^1^, Elena Cantone^2^, Antonella M. Di Lullo^2,4^, Marcella d’Ippolito^1^, Assunta Celardo^1^, Lucia Visaggi^1^, Maurizio Di Cicco^3^, Maurizio Iengo^2^, Giuseppe Castaldo^4^, Vincenzo Carnovale^1^

##### ^1^Regional Center of Cystic Fibrosis, Adult Unit, Department of Medical Translational Science, University of Naples “Federico II”, Naples, Italy; ^2^Department of Neuroscience, ENT Section, University of Naples “Federico II”, Naples, Italy; ^3^ORL Cystic Fibrosis Center, UO ORL, IRCCS “Fondazione Policlinico Ca’ Granda” Milan, Milan, Italy; ^4^CEINGE- Advanced Biotechnology, Naples, Italy

###### **Correspondence:** Serena Buonaurio (serenabuonaurio@libero.it)

**Background**

Cystic Fibrosis (CF) presents multiorgan manifestations that include chronic rhinosinusitis (CRS) with or without nasal polyposis. Nasal washes (NWs) are widely used in clinical practice especially in CF patients, although their effectiveness on Ear Nose Throat (ENT) symptoms is controversial.

In this study we evaluate the performance and the safety of a NWs solution, with or without surfactant, to reduce symptoms and bacterial load.

**Materials and methods**

We enrolled 20 CF patients (mean age: 27.6 years) with CRS, confirmed by nasal endoscopy. All patients, colonized by Pseudomonas Aeruginosa, performed daily a NW by physiological solution or by saline solution with surfactant (Naridek). All patients, at the time of enrollment, filled out a sinonasal questionnaire (SANQ11) and they received instructions for proper washing. During follow-up, we evaluated the reduction of the bacterial load in the nasal lavage. We assessed the nasal cavities by endoscopy (2.7 mm 30° rigid endoscope - Storz, Tuttlingen, Germany) according to a modified Lund Kennedy endoscopic scoring system:

- rhinorrhea (present = 0, mild = 1, purulent = 2);
- edema, and hyperemia (absent = 0, mild = 1 or severe =2)
- nasal mucosa (eutrophic = 1; hyperemic = 2; dystrophic = 3);
- left and right turbinate hypertrophy (none = 0; mild = 1; medium = 2; and serious = 3).

All subjects underwent the Sniffin’Sticks to evaluate the olfactory performance.

**Results**

Twelve patients completed 4 months of treatment: 6 patients performed the treatment with Naridek and 6 patients with physiological solution.

Due to the small sample size, the scores were added together to have an overall indication of the treatment (Table 1)

The bacterial colonization in NWs shows no statistically significant difference. However, in 2 patients, we detected a reduction of the bacterial load, while there was no difference in the saline-treated group.

**Conclusions**

Considering our small sample we can only draw some great deal to think about:

- treatment with NWs allows an improvement of the ENT symptoms and is well tolerated by patients. These data are confirmed by the ENT signs score and by the reduction of the SNAQ11 score in both treatment arms;
- the solution with surfactant (Naridek) significantly improves the ENT signs and decreases the nasal endoscopy and the SNAQ11 scores;
- no benefit was detected at the evaluation of olfactory performance.

In conclusion, even if further confirmations are necessary on broader cases, it seems to emerge as significant the role of surfactant in the therapeutic advantage of NWs.

**Table 1 (abstract A36).** Results

	Nasal endoscopy	ENT signs score	Olfactory performance	SNAQ 11
Naridek V1	80	72*	169	197**
Naridek V4	15*	15*	159	98**
Physiological solution V1	68	61	151	166
Physiological solution V4	52	39	159	152

*P<0.05 ** p< 0,01

### A37 Burkholderia gladioli: a new potential hazard in Cystic Fibrosis patients?

#### Daniela Dolce^1^, Mattia Giovannini^2^, Gilda Belli^2^, Novella Ravenni^1^, Silvia Campana^1^, Tommaso Orioli^1^, Cesare Braggion^1^, Giovanni Taccetti^1^

##### ^1^Cystic Fibrosis Center, Interdisciplinary Specialist Department, Anna Meyer Children's University Hospital, Florence, Italy; ^2^Department of Health Science, University of Florence, Anna Meyer Children's University Hospital, Florence, Italy

###### **Correspondence:** Daniela Dolce (d.dolce@meyer.it)

**Background**

*Burkholderia gladioli* is an opportunistic Gram-negative, not belonging to *Burkholderia cepacia* complex (BCC). Its clinical impact ranges from transient to chronic infections and it is responsible of poor post-lung transplant outcomes. Therapeutic strategies are not standardized. We describe 5 cases of *B. gladioli* infection in cystic fibrosis (CF) patients to highlight the wide variability in the clinical course.

**Materials and methods**

Presumptive identification of *B. gladioli* was assessed by MALDI-TOF and confirmed by 16S sequencing. The isolates from single patients and stains isolated from persistently infected patients were genotyped both with BOX-PCR approach and MLST analysis.

**Results**

*B. gladioli* was isolated in 5 (1.6%) out of 305 patients attending our Center:

1. P1 is a 45-years-old female, diagnosed with CF at the age of 8. At the age of 36, *B. gladioli* was isolated from sputum. Despite various antibiotic treatments the pathogen was persistently isolated and we observed a relevant FEV_1_ decline.
2. P2 is a 48-years-old male, diagnosed with cystic fibrosis at 20. At the age of 37, *B. gladioli* was isolated. He developed chronic *B. gladioli* infection and underwent a progressive clinical deterioration.
3. P3 is a 13-years old patient diagnosed by newborn screening. At the age of 8 years a reduction in pulmonary function was detected and *B. gladioli* was isolated in the sputum. Antibiotic treatment was associated with *B. gladioli* clearance.
4. P4 is a 3- years-old female. At the age of 3 she underwent sinuses surgery (FESS). *B. gladioli* was isolated in the nasal lavage. Normal flora from upper and lower respiratory tract was found during the follow-up.
5. P5 is a 28-years-old male, diagnosed by newborn screening. At the age of 28, *B. gladioli* was isolated in respiratory samples. Further microbiological evaluations revealed spontaneous clearance of *B. gladioli* from the respiratory tract.

We genotyped strains from 4 out of 5 patients. The isolates were genetically unrelated. Instead, strains isolated from each chronically colonized patient showed an identical profile. MLST profiles showed the environmental origin of the bacteria.

**Conclusions**

Sometimes *B. gladioli* may cause transient infections, especially if strains are susceptible to antibiotics. Cases of chronic infections have been reported and antibiotic strategies are not clearly established. In our study genotyping showed that all patients were infected with genetically distinct strains, while a single strain was responsible of persistent infection in two cases. Large multicenter studies are needed to investigate epidemiology, clinical course and effect of *B. gladioli* on lung disease in CF.

Written informed consent for publication was received from the patient/parent of the patient

### A38 Ceftazidime–Avibactam and Ceftolozane/Tazobactam: new antimicrobials against multidrug-resistant *Pseudomonas aeruginosa* in Cystic Fibrosis

#### Daniela Dolce, Novella Ravenni, Silvia Campana, Erica Camera, Cesare Braggion, Giovanni Taccetti

##### Cystic Fibrosis Center, Interdisciplinary Specialist Department, Anna Meyer Children's University Hospital, Florence, Italy

###### **Correspondence:** Daniela Dolce (d.dolce@meyer.it)

**Background**

The emergence of multidrug-resistant *Pseudomonas aeruginosa* (MDR-PA) strains is an important and growing issue in the care of cystic fibrosis (CF) patients. Ceftazidime–avibactam and ceftolozane/tazobactam are novel antimicrobials combining a third-generation cephalosporin with β-lactamase inhibitor the use of which are restricted to some clinical cases, including CF patients infected with MDR-PA. In this study, we assessed the ceftazidime–avibactam and ceftolozane/tazobactam susceptibility of MDR-PA isolated from adults with CF.

**Materials and methods**

MDR-PA (strains resistant to all antibiotics in two or more classes of antipseudomonal agents), were isolated from CF patients in our center in the last year. The antimicrobial susceptibility for all the isolates were performed by using the micro broth dilution method following EUCAST guidelines.

**Results**

The activity of ceftazidime/avibactam and ceftolozane/tazobactam was assessed against 29 MDR-PA isolates collected from 15 CF patients (mean age 34.0±11.0 yrs) chronically infected. We also analysed 32 strains from a control group of 12 CF patients (mean age 29.7±9.8yrs) with chronic no-MDR *Pseudomonas aeruginosa* infection. The rate of susceptibility of MDR isolates to colistin and ceftazidime/avibactam were 100% and 48.3% respectively, followed by 27.6% to ceftolozane/tazobactam (MIC_50/90_, 16/32 mg/liter) and tobramycin. Ceftazidime-avibactam (MIC_50/90_, 4/16 mg/liter) was the most active (highest susceptibility rates) compounds after colistin. All other antibiotics were active only in less than 10% of the isolates, no isolates were susceptible to ciprofloxacin. In the non-MDR isolates the susceptibility rates were 96.9% and 90.6% to ceftolozane/tazobactam (MIC_50/90_, 1/2 mg/liter) and ceftazidime-avibactam (MIC_50/90_, 2/8 mg/liter) respectively. All antimicrobials resistance rates are shown in the table 1.

**Conclusions**

Ceftazidime–avibactam and ceftolozane/tazobactam are novel antimicrobial drugs targeting difficult to treat Gram-negative organisms. The data showed that these antimicrobials were highly active *in vitro* against CF isolates of *Pseudomonas aeruginosa*, including isolates that exhibit resistance to multiple drug classes. This study demonstrates excellent susceptibility profiles specially for ceftazidime–avibactam against MDR *Pseudomonas aeruginosa* strains collected from the sputum of individuals with CF.

**Table 1 (abstract A38).** Pa antimicrobials resistance rates

	GEN	AMI	TOB	CIP	CAZ	AZT	IMI	MEM	TZP	CZA	C/T	COL
Pa MDR % S	3.7	3.4	27.6	0.0	10.3	6.9	10.3	17.2	13.8	48.3	27.6	100.0
Pa no-MDR % S	65.6	75.0	87.5	21.9	81.3	31.3	59.4	31.3	15.6	90.6	96.9	100.0

**Abbreviations:** Pa, *Pseudomonas aeruginosa*; MDR, Multidrug-resistant; GEN, Gentamicin; AMI, Amikacin; TOB, Tobramycin; CIP, Ciprofloxacin; CAZ, Ceftazidime; AZT, Aztreonam; IMI, Imipenem; MEM, Meropenem; TZP, Piperacillin / tazobactam constant 4; CZA, Ceftazidime/avibactam; C/T Ceftolozane/tazobactam 4; COL, Colistin

## RESPIRATORY

### A39 Cystic Fibrosis Sinus Score for paranasal sinuses complications of Cystic Fibrosis: a three-years long experience

#### Maurizio Di Cicco^1^, Daniele Di Pasquale^1^, Giovanna Pizzamiglio^2^, Carla Colombo^2^

##### ^1^ENT Department, Foundation Cà Granda, IRCCS Policlinico Hospital, Via F. Sforza 35, 20122, Milan, Italy; ^2^Regional Referral Centre for Cystic Fibrosis, Foundation Cà Granda, IRCCS Policlinico Hospital, Via F. Sforza 35, 20122; University of Milan, Milan, Italy

###### **Correspondence:** Maurizio Di Cicco (maurimdc@yahoo.com)

**Background**

Management of paranasal sinuses complications in Cystic Fibrosis (CF) is a challenge for the otolaryngologist. Paranasal sinuses system represents *a P. aeruginosa* reservoir affecting lower airways system. Early diagnosis and follow-up of ENT complications of CF, like mucoceles and polyposis, are important to treat them. A clinical- symptoms, radiological score for treatment, stratification and evaluation of CF patients has been developed.

**Materials and methods**

Between May 2015 and May 2018, 52 patients (27 males, 25 females; mean age 15.90 years, range 6-30 years) attending our ENT-CF clinic were evaluated for paranasal sinuses complications with maxillo-facial CT; cone-beam CT (CBCT) investigation was used for pediatric patients. Radiological results were described qualitatively and quantitatively using Lund-Mackay score. CT and CBCT report with the radiological score, endoscopic Meltzer's Score and SNAQ-11 questionnaire for clinical ENT symptom were used for the assessment of a CF sinus score (CFSS), with a maximum score of 112 points. ENT treatment was chosen depending on CFSS results.

**Results**

CT and CBCT guided Lund-Mackay scores: mean result 20.25 (range 6-24). By CFSS findings, 20 patients (38%) were addressed to endoscopic sinus surgery (mean result 67.5; range 32-80); the remaining patients were addressed to follow-up/medical therapy (mean result 23.8; range 12-42). Most relevant radiological findings were pluri-sinonasal disease, medialization of maxillary sinuses walls, mucoceles, and bone resorption. At endoscopic evaluation polyposis was the most frequent finding (61.5%); finally, nasal obstruction and rhinorrhea were registered as the most referred symptoms (42% and 21% each).

**Conclusions**

Our CFSS results confirmed that CF patients present an early clinical objectivity of nasal polyposis with pluri-sinonasal disease, asymptomatic in most of the cases. CBCT has demonstrated to be a suitable radiological investigation and follow-up for CF patients, due to its reduced radiation exposition. Future objectives of CFSS is to identify CF patients who can benefit the most from surgery and to become a mean for comparison among ENT specialists treating CF.

Went to our referred center complaining dyspnea at rest, worsening of airway clearance and the need for more oxygen support during the day.

### A40 High flow nasal cannula for exacerbation of dyspnea in patient with Cystic Fibrosis: a case report

#### Francesca Nori^1^, Giacomo Vandi^2^, Elena Balestri^2^, Stefania Dall’Ara^2^, Alessandro Valentino^1^, Fiorella Battistini^2^

##### ^1^Dipartimento Chirurgico e Grandi Traumi, Ospedale M. Bufalini Cesena, Cesena FC, Italy; ^2^SSA Servizio Fibrosi Cistica, Centro di riferimento Regionale, Ospedale M. Bufalini Cesena, Cesena FC, Italy

###### **Correspondence:** Francesca Nori (francesca.nori@gmail.com)

**Background**

We presented the case of a 27-yr-old man with Cystic Fibrosis (CF) due to a DF508 homozygotic mutation. The patient is colonized with Pseudomonas aeruginosa, Aspergillus fumigatus and Mycobaterium ascessus that were treated in the past with no evidence of reactivation, and an allergic bronchopulmonary aspergillus (ABPA) was detected. He is currently treated with a CFTR corrector (lumacaftor) and a CFTR potentiator (ivacaftor). Patient was treated with low oxygen flow (LOF) support continuously throughout the day. Before the admission, the health perception was assessed according to CFQ-R Teen/Adult Italian Version 2.0. The patient went to our referred center complaining dyspnea at rest, worsening of airway clearance and the need for more oxygen support during the day.

**Materials and methods**

Patient was admitted with high respiratory rate (35 breaths/min). His first gas assessment showed hypoxemia (pO2 55.8 mmHg) and low pO2/FiO2 ratio (1.36). Chest X-ray was suggestive for a new opacity. Then, high flow nasal therapy (HFNC) (AIRVO 2 Fisher and Paykel) was administered to the patient (34 °C, 50 L/min and FiO2 42%). Patient continued high flow nasal cannula therapy almost continuously for seven days and for a further four nights. We prescribed HFNC domiciliary at night time.

**Results**

Patient reported great comfort and showed high tolerance to this device and setting. Respiratory rate decreased shortly after the start of high oxygen flow therapy. Gas assessment at the end of treatment with HFNC revealed increased level of pO2 and pO2/FiO2 (pO2 85.9 mmHg, PO2/FiO2 2.45). The patient acknowledged an improvement in cleaning the secretions. At the time of discharge, the patient pointed out the great benefits of the HFNC treatment and his general health perception increased according to CFQ-R Teen/Adult Italian Version 2.0.

**Conclusions**

In patients with CF the clearance of airway secretions remains one of the most common issues. It is well known that HFNC delivers warm and humidified gas and thus improves mucociliary function by maintaining clearance to a greater degree than other methods for delivering oxygen. Due to the capacity of the device to reach higher and relatively fixed FiO2 than LOF, hypoxemia improves throughout the treatment. Furthermore, HFNC generates positive end-expiratory pressure which reduces the work of breathing and respiratory rate. The low burden of the device allows the patient to continue the treatment also at home with potential benefits in terms of health perception, cleaning secretions and respiratory physiotherapy.

The patient gave the consent to publish clinical data.

### A41 Observational study of sleep respiratory breathing disorders and preventive noninvasive ventilation in cystic fibrosis: preliminary results and proposal of longitudinal and multicenter study

#### Maria Papale, Giuseppe F Parisi, Chiara Franzonello, Lucia Tardino, Enza Mulè, Novella Rotolo, Salvatore Leonardi

##### UOC Broncopneumologia Pediatrica e Fibrosi Cistica – Università degli Studi di Catania, Catania, Italy

###### **Correspondence:** Maria Papale (mariellapap@yahoo.it)

**Background**

In Cystic fibrosis (CF) lung disease is characterized by progressive airflow obstruction, due to mucus plugging and airway inflammation, causing destruction of the lung parenchyma secondary to bronchiectasis. These alterations result clinically in an increase of the work of breathing, leading to alveolar hypoventilation predominantly during sleep, exercise and acute respiratory exacerbations. In clinical practice, nocturnal cardiorespiratory polygraphy (PG) role is growing and may represent an important diagnostic test compared with conventional pulmonary function test as spirometry and lung clearance index (LCI). Furthermore, signs of lung function decline, marked principally by impaired gas exchange and/or increased work of breathing during sleep, may be represent powerful and validated criteria to start preventive nocturnal noninvasive ventilation (NPPV) in CF patients. The purpose of this study was to identify respiratory patterns over the spectrum of disease severity in patient with CF. The overall hypothesis for the current study is that patients with CF demonstrate gas exchange abnormalities and increased respiratory loads during sleep, reported and recognized by conventional cardiorespiratory PG and also, using non-invasive ventilation as preventing respiratory failure in contrast with conventional practice in clinical studies.

**Materials and methods**

Analysis of nocturnal breathing patterns and gas exchange on PG was performed in patients with CF (30) patients with different clinical characteristics. They performed also spirometry and LCI with multiple-breath washout (MBW) test.

**Results**

Children with CF demonstrated lower oxyhemoglobin saturation (95% ± 1.6%), higher respiratory rate (20.5 ± 6.9 breaths per minute). The Apnea Hypopnea index differ between age, particularly in preschool age (5.5 ± 2.7 events per hour,). Ventilation distribution outcomes (LCI,) was significantly higher (>7) in patients with advanced CF. 5 patients with serious nocturnal hypoxemia and/or increasing respiratory loading started nocturnal noninvasive Bi-level ventilation. After 6 months, control polygraphy showed an improvement of respiratory pattern.

**Conclusions**

NPPV may be represent a potential treatment in CF to prevent lung function decline, in contrast with conventional use in acute respiratory failure, or as a bridge to pulmonary transplant. Using Bi-level PPV, upper airway obstruction and/or work of breathing induced by intrinsic positive pressure of end expiration (PEEP) are prevented by expiratory positive airway pressure (EPAP) and thus pression support (PS) can be triggered easily by the patient. In conclusion, different ventilatory modes have been used in patients with CF, but bi-level positive pressure ventilation is used by the majority of the patients for the comfort and the security regarding to the inspiratory pressures.

### A42 ECMO as a bridge to recovery from severe exacerbation in non-end stage lung disease of a patient with Cystic Fibrosis: a case report

#### Riccardo Guarise^1^, Beatrice Borchi^1^, Silvia Bresci^1^, Annalisa Cavallo^1^, Manuela Bonizzoli^2^, Chiara Lazzeri^2^, Giovanni Cianchi^2^, Adriano Peris^2^, Cesare Braggion^3^, Alessandro Bartoloni^1^

##### ^1^Tropical and Infectious Disease Department, Careggi, Florence, Italy; ^2^Anaesthesia and Intensive Care Unit of Emergency Department, Careggi, Florence, Italy; ^3^Cystic Fibrosis Center, Anna Meyer Children's Hospital, Florence, Italy

###### **Correspondence:** Riccardo Guarise (guariser@aou-careggi.toscana.it)

**Background**

Extracorporeal life supports (ELS) are devices that enable direct oxygenation or CO_2_ extraction from the blood. In recent years their use has increased among CF patients with end-stage respiratory condition and failure as a bridge for lung transplantation (LTx). Nevertheless, extracorporeal membrane oxygenation ECMO support (ECMO) is considered when a patient presents a rapid deterioration of a chronic lung disease and only when it has been already included on a LTx waiting list or an evaluation process has already started.

**Case Report**

A 25 years old woman with CF, CF-related diabetes (CFRD), CF liver disease (CFLD) with portal hypertension and a history of moderate but unstable lung disease, chronic Pseudomonas Aeruginosa colonization and allergic bronchopulmonary Aspergillosis (ABPA) presented with fever (38°C), persistent cough, excessive stagnation of thick mucus and dyspnea. Chest X-ray showed diffuse bilateral parenchymal thickening, several bronchiectasis with mucoid impaction, presence of voluminous sub-pleural bubbles. Intravenous antibiotics and corticosteroids were started along with intensive regimen of respiratory physiotherapy for airway clearance.

During the first hours her status deteriorated, so that she needed to be transferred to the intensive care unit (ICU) where she required intubation and invasive mechanical ventilation (IMV) with assisted pressure support mode. After 4 hours, despite IMV at maximal protective pressure and maximal antibiotic coverage, arterial blood gas (ABG) analysis showed a persistent unresponsive hypoxaemia, severe respiratory acidosis and severe hypercapnia (pH 7.18, pCO_2_ 87 cm H_2_O), so veno-venous ECMO was initiated. After 40 hours, ABG analysis improved, so she was successfully extubated and placed on continuous NIV. During ECMO period, mucolytic therapy was doubled and two sessions per day of airway clearance were performed by respiratory physiotherapists. Ventilation parameters improved, so she has been “bridged” from NIV to heated humidified high-flow nasal cannula therapy. ECMO de-cannulation occurred on day 10 and after 48 hours she was discharged from ICU on spontaneous breathing with 5 lt/min oxygen supplementation. Respiratory physiotherapy was continued, reassessed and optimized along with a training program. She was discharged home on after 10 days without oxygen supplementation.

**Conclusion**

Considering clinical history and the stage of pulmonary disease, this patient wasn’t considered yet for LTx. ECMO is an important support to severe CF exacerbations unresponsive to conventional treatments in patients that are not yet candidates to LTx. ECMO “bridge-to-recover” use is emerging in CF and could extend native lungs function in order to take time for waiting list evaluation and optimize allocation.

The patients gave the consent to publish clinical data.

**Table 1 (abstract A42).** Arterial Blood Gas analysis, Mechanical Ventilation and ECMO parameters during the ICU hospitalization

Time from admission	pH	pO2 [mmHg]	pCO2 [mmHg]	SaO2 [%]	FiO2 [%]	IPAPIPA sB [cm H2O]	EPAP [cm H2O]	HFNC F/ow [Ltlmin]	Blood Flow [Ltlmin]	Gas Flow [Ltlmin]
0 hr					Start of /MV					
1 hr	7,18	141,0	87,9	98,8	100	28	8			
4 hr					Start of ECMO					
6 hr	7,40	95,7	50,0	98,3	25	25110	8		2,92	2
10 hr	7,44	68,7	45,0	94,5	30	23110	8		2,9	2,5
38 hr	7,50	77,5	45,6	96,2	35	22116	8		3,47	3
41 hr					End of /MV and start of NIV					
42 hr	7,51	87,0	44,7	97,6	42	10	7		3	3
48 hr	7,52	67,4	42,7	94,3	40	12	7		3,56	5
60 hr	7,55	88,2	36,8	98,0	40	14	6		3,8	5
99 hr				End of nocturnal NIV and start of diurna/ HFNC						
100 hr	7,42	88,0	47,4	96,7	60	12	7	40	2,8	5
110 hr				End of diurna/ NIV and start of nocturnal HFNC						
120 hr	7,50	68,8	34,7	94,3	60			40	2,73	7
144 hr	7,50	64,6	35,0	93,0	50			30	2,54	6
162 hr	7,47	74,2	34,3	95,0	50			30	2,9	5
182 hr	7,47	100,0	34,4	98,2	45			30	2,26	4
191 hr	7,47	94,7	35,8	97,6	46			30	1,8	2,5
207 hr					Start ECMO OFF Tria/					
212 hr	7,44	70,3	37,2	94,0	50			20	1,87	2,5
217 hr	7,44	88,7	40,8	96,8	45			20	1,98	3,5
218 hr					End of ECMO OFF Tria/					
231 hr	7,45	103,0	36,6	98,2	60			20	2,37	3,5
232 hr					End of ECMO					
271 hr	7,44	80,3	51,6	96,4	50			20		

/PAP: lnspiratory Positive Airway Pressure, EPAP: Expiratory Positive Airway Pressure, PAsa: Pressure at assisted spontaneous breathing, HFNC: High- F/ow Nasal Cannu/ae, /MV: Invasive Mechanica/ Ventilation, NIV: Non-invasive ventilation

## PHYSICAL AND RESPIRATORY THERAPY

### A43 Comparing the efficacy of two different training modality in adult patients with cystic fibrosis: a retrospective observational study

#### Luigi Graziano^1^, Tamara Perelli^1^, Marco Martini^2^, Daniele Biacchi^3^, Daniela Savi^1^, Giuseppe Cimino^1^, Marco Rivolta^1^, Serenella Bertasi^1^, Enea Bonci^4^, Paolo Palange^1^

##### ^1^Cystic Fibrosis Centre, Policlinico Umberto I, Rome, Italy; ^2^Sapienza University of Rome - Physiotherapy BSc, Rome, Italy; ^3^Department of Surgery “P. Valdoni”, Sapienza University of Rome, Italy; ^4^Department of Experimental Medicine, Sapienza University of Rome, Italy

###### **Correspondence:** Luigi Graziano (luigi.graziano@uniroma1.it)

**Background**

It is well established that exercise has multiple benefits for people with cystic fibrosis (CF), but the evidence for health effects of specific sport disciplines is poor. Therefore, we retrospectively examined the impact of two different training modality - jumping on a trampoline and cycling on a stationary bike - on spirometry, exercise capacity measured with 6 Minute Walk Test (6MWT), exertional dyspnea and the perception of muscular fatigue.

**Materials and methods**

A six months retrospective observational study was conducted from 1st March to 31st August 2018 at the Cystic Fibrosis Centre, Policlinico Umberto I of Rome (Italy). Data were collected from medical records of 30 adult CF patients (19 F; mean age 31.1 ± 10.2 yrs) hospitalized for pulmonary exacerbation (mean hospital stay 14.2 ± 3.5 days). The main outcomes were spirometric values (FVC, FEV_1_, FEF_25-75_), secondary outcomes were 6 minutes walked distance, dyspnoea (measured with a Borg scale before and after the 6MWT) and muscular fatigue perception measured in the same way. A comparative analysis was conducted to test the significance of differences within same group and between groups. SPSS version 24.0 was used for data entry and statistical analysis. Univariate comparisons were made using Student's t-test. Alpha was set at 0.05.

**Results**

Participants of both groups had improvement in all outcomes, presumably due to the overall treatment received. The study shows no statistically significant difference between groups for the main outcome while the 6 minutes walked distance (from 566.1 ± 47.0 m to 604.7 + 43.8 m; p=0.001), dyspnoea (from 6.5 ± 1.5 to 3.7 + 1.3; p=0.004) and muscular fatigue perception (from 3.8 ± 1.5 to 1.7 + 0.7; p=0.001) have improved in the trampoline group in a statistically meaningful way. The retrospective design of the study did not allow to know patients’ preference between jumping and cycling.

**Conclusions**

Jumping on a trampoline could be a valid training modality for people with CF, but more research is needed to better understand its efficacy, safety and patients preference compared with other training modality.

### A44 Manual therapy for musculoskeletal pain in cystic fibrosis: review of the literature

#### Luigi Graziano^1^, Barbara Giordani^2^, Daniela Nico^3^, Elia Colella^3^, Daniela Savi^1^, Giuseppe Cimino^1^, Alessandro Di Vito^4^, Donatella Valente^5^, Paolo Palange^1^

##### ^1^Cystic Fibrosis Centre, Policlinico Umberto I, Rome, Italy; ^2^Lega Italiana Fibrosi Cistica ONLUS - LIFC, Rome, Italy; ^3^Sapienza University of Rome - Physiotherapy BSc, Rome, Italy; ^4^San Giovanni Addolorata Hospital, Rome, Italy; ^5^Department of Human Neuroscience, Sapienza University of Rome, Rome, Italy

###### **Correspondence:** Luigi Graziano (luigi.graziano@uniroma1.it)

**Background**

Pain is a common complication in patients with cystic fibrosis (CF), negatively associated with pulmonary exacerbations, quality of life and treatment adherence. Moreover, with the increased life expectancy the prevalence of musculoskeletal complications is growing, and the use of physiotherapy techniques to minimise these problems has increased. The aim of this study is to review the efficacy of physiotherapeutic manual techniques to manage musculoskeletal pain in patients with CF.

**Materials and methods**

MEDLINE, CINAHL, PsycINFO, Scopus, CFDB and trials registries via WHO Portal were searched since 2000 up to September 14t 2018. Search terms included (cystic fibrosis) AND (musculoskeletal pain) AND (manual therapy) AND (sham therapy OR rest OR exercise OR drug OR surgery).

**Results**

364 studies were identified, of which 4 (190 participants) met the inclusion criteria: 3 randomised controlled studies (85 participants) and one non-randomized study (105 participants). The study size ranged from 20 to 105 participants (age 18 or older). Duration of treatment in the included studies ranged from one day to six months. Due to data heterogeneity no meta-analysis could be performed. Manual therapy (in the form of osteopathic manipulative treatment, spinal joint and intercostal mobilization, soft tissue therapy, massage or chiropractic) was compared with sham therapy or conventional care (no manual therapy). Three RCT compared manual therapy with sham therapy or no treatment (usual care). Primary endpoints were FEV_1_ and a composite outcome reflecting the intensity of pain and the number of painful days over the previous month. Secondary endpoints were self-assessment of breathing, pain and anxiety level measured with a questionnaire, quality of life (Cystic Fibrosis Questionnaire), need for analgesics. The studies showed no statistically significant difference between the treatment groups and the control groups, it is however interesting to note that in one study 26 patients (81%) indicated in a questionnaire that they had been interested in the study and 15 of 16 patients (94%) in the treatment arm were very satisfied with the treatment received. One non-randomized trial studied the effect of a combination of musculoskeletal physiotherapy techniques and massage therapy (a single treatment session) on musculoskeletal pain and ease of breathing (VAS) showing a significant reduction in both outcomes, irrespective of clinical status (acute versus stable patients).

**Conclusions**

Larger and more powerful studies are needed to determine whether manual therapy techniques could be a valuable therapeutic option in the management of musculoskeletal pain for patients with cystic fibrosis. Patients interest and satisfaction also encourage further research.

### A45 Intrapulmonary percussive ventilation with high-frequency chest wall oscillation: make way for positive vibration? A case report

#### Marco Rivolta, Luigi Graziano, Tamara Perelli, Daniela1Savi, Giuseppe Cimino, Dario Asciutti, Serenella Bertasi, Paolo Palange

##### Cystic Fibrosis Centre, Policlinico Umberto I, Rome, Italy

###### **Correspondence:** Marco Rivolta (marco.rivolta94@gmail.com)

**Background**

Six-year-old girl admitted in the Cystic Fibrosis Centre, “Policlinico Umberto I” of Rome, for a pulmonary exacerbation colonized with Pseudomonas Aeruginosa. She had a CT thorax: various bronchiectasis filled with mucus in lingular area, in superior right lobe and superior left lobe. During hospitalization she received antibiotic therapy, corticosteroids, dornase alpha and, in the first days, daily respiratory physiotherapy with pep-mask. Before the admission, the patient used to have dance classes, four times a week and jumped on a trampoline every day for 45 minutes. The patient’s adherence was good - her family actively participated in treatment plan - but her attitude towards the cure, physiotherapy in particular, was critical, because the girl perceived the therapeutic burden as very high.

**Materials and methods**

In an attempt to increase efficacy and the girl appreciation for treatment, during the 14 days hospitalization she received the prescribed treatment plan together with a new physiotherapy modality using at the same time the “High frequency chest wall oscillation” (Respin 11 Bronchial Clearance System®, Respin-USA Inc. Clarksburg (MD) USA) and the “Intrapulmonary percussive ventilation” (IPV® Percussionaire Corp. (ID) USA), twice a day, each session lasting 30 minutes. The program used for Respin 11® system was “Minnesota kids”. We used three modalities for IPV®, each one lasting 9 minutes: middle flow rate, high frequency; middle flow rate, middle frequency; middle flow rate, low frequency. During the one minute break between the modalities, the patient performed assisted coughing. Furthermore, she had a 40 minutes playful physical activity session.

**Results**

At discharge, there was an improvement of the patient’s clinical status: less rattlings, good lung air penetration. Although we couldn’t use any validated evaluation scale, there are several evidences that she liked this new physiotherapy treatment: the girl’s teacher showed us some draws of her having physiotherapy, her parents reported she was happy during the treatment so the perceived treatment burden decreased.

**Conclusions**

Medical treatments improve patients’ clinical status, but submitting them to a heavy treatment burden. Hence, it is important to find a way to make the treatment more appreciated. In this patient, a non-conventional tool has proved to be successful: the girl changed her attitude to positive, by showing happiness during physiotherapy. This could be an important aspect we could deepen to increase adherence in pediatric patient subjected to heavy treatment burden.

The patient gave the consent to publish clinical data.

### A46 Tracking adherence through I-Neb: you never stop learning!

#### Alessandra Mariani^1^, Anna Brivio^1^, Simone Gambazza^1,2^, Federica Carta^1^, Mara Bergamini^1^, Elvira Caverni^1^, Carla Colombo^1,3^

##### ^1^Fondazione IRCCS Ca’ Granda, Ospedale Maggiore Policlinico, Cystic Fibrosis Centre, Milan, Italy; ^2^Università degli Studi di Milano, Dipartimento di Scienze Cliniche e di Comunità, Milan, Italy; ^3^Università degli Studi di Milano, Dipartimento di Fisiopatologia Medico-Chirurgica e dei Trapianti, Milan, Italy

###### **Correspondence:** Alessandra Mariani (alessandra.mariani@policlinico.mi.it)

**Background**

Time requirements for multiple daily nebulizer treatments are important impediments to the quality of life for most patients with cystic fibrosis (CF). The I-neb Adaptive Aerosol Delivery (AAD) results in shorter treatment times and better deposition of the drug system, if used correctly. It can be used with 2 modality of breathing: the Target Inhalation Mode (TIM) or Tidal Breathing Mode (TBM). Most importantly, it allows patients, care-giver and CF team to have feedback on the ongoing aerosol therapy. The aim of this study was to evaluate the need of an educational intervention (EI), assessed by recorded adherence, inhalation technique and mesh performance on I-neb device.

**Materials and methods**

The platform *Insight Online* (IoL) used by respiratory physiotherapists at the CF Centre of Milan was initially screened to detect patients actively on I-neb. Adherence, inhalation and nebulization time, and cleaning data were reviewed and analysed.

**Results**

After the initial screening, some data were dropped because withdrawal (n=22), patient changed CF center (n=1) and early termination (n=14). There were 43 patients actively on inhaled antibiotics treatment with I-neb on IoL. To date, 30 patients with CF aged 5-34 yrs were considered for the analysis (13 have not been followed-up yet): 27 used TIM and 3 the TBM breathing modality. Overall, I-neb compliance was 72.4 (36.1)% (range 0 to 132%); the mean rest time was 37.7 (31.4)% (range -6 to 88%) and the mesh performance was 78.2 (40.3)% (range 0 to 147%). The mean nebulization time when using TIM was 2’5’(2’3”) (range 0”to 8’1”); if TBM was used, the mean time was 8’0”(4’5”) (range 4’6”to 13’05”). Mean inhalation time per breath using TIM was 2,8”(2,7”) (range 0” to 7,9”), using TBM was 1’0”(0,9”) (range 0,4” to 2,1”).

**Conclusions**

IoL gives a unique opportunity to monitor patients’ adherence, the performance of the aerosol device and can provides information that could have been missed if using self-reporting. On average, the majority of patients showed good results, however there is a great variability when using this device. Therefore, it may be necessary to provide an EI to train patients at inhaling. It may be also useful to make the training software available at home to improve the inhalation technique.

**References**

1. Denyer J et al. Journal of Aerosol Medicine and Pulmonary Drug Delivery. 2010; 23(Suppo): s45-54

2. Donà M et al. Journal of Cystic Fibrosis. 2013; 12 (Suppl): s105

3. Button BM et al. Respirology. 2016; 21:656-67.

### A47 Evaluating physical performance in paediatric patients: experience of the Cystic Fibrosis (CF) centre of Milan

#### Anna Brivio^1^, Simone Gambazza^1,2^, Anna Maria Clotilde Bulfamenta^1^, Alessandra Mariani^1^, Federica Carta^1^, Mara Bergamini^1^, Elvira Caverni^1^, Carla Colombo^1,3^

##### ^1^Fondazione IRCCS Ca’ Granda, Ospedale Maggiore Policlinico, Cystic Fibrosis Centre, Milan, Italy; ^2^Università degli Studi di Milano, Dipartimento di Scienze Cliniche e di Comunità, Milan, Italy; ^3^Università degli Studi di Milano, Dipartimento di Fisiopatologia Medico-Chirurgica e dei Trapianti, Milan, Italy

###### **Correspondence:** Anna Brivio (anna.brivio@policlinico.mi.it)

**Background**

Standardized exercise is part of the regular assessment of patients with CF [1]. It is used for the evaluation of physical limitations, to document exercise-associated symptoms or to screen for possible adverse effects of exercise [2]. Exercise is an important therapeutic modality for individuals with CF, with higher physical activity levels being associated with greater aerobic fitness [3], pulmonary function, glycemic control [4], and bone mineral density. The Godfrey protocol has been used for the clinical assessment of adverse reactions to exercise in CF. The aim of this study is to evaluate exercise tolerance in paediatric patients regularly followed-up at Milan CF centre.

**Materials and methods**

After reviewing the literature and the feasibility of different exercise testing at our centre, we adopted the Godfrey cycle protocol since November 2014 as routinary assessment of exercise. Informed consent and test operator procedure were then realized. After March 2018, following the donation of a paediatric cycloergometer, we have been testing the paediatric population below 120 cm-height as well.

**Results**

So far**,** 63 patients (31 female) with CF have performed the Godfrey test. Mean (SD) age was 13.8 (2)yrs (range 7.8 to 15.9 yrs) with a FEV_1_ of 85 (26.2)%pred. 4 patients (6.3%) had FEV1 <40%pred), BMI perc 39,39°(26.5); any tests have been performed with oxygen therapy.

HR peak was 171.72 (14.8) beats/min, HR percentage was 88.3% (7.7), Wmax was 122(42.8) watt. Only 35 tests (55.5%) were maximal: 15 for Wmax, 27 for BORG CR10 ≥ 9, 7 for HR peak. Only 7,5% (n=12) of the whole sample showed a normal response to exercise. The average time needed for the test was 10’08” (6’23” to 14’25”).

**Conclusion**

These data suggest that in the pediatric population exercise testing offers an opportunity of detecting significant abnormalities. It would be important to perform cardio-pulmonary exercise testing in patients with abnormal response.

**References**

1. Hebestreit H, Arets HG, Aurora P et al for the European Cystic Fibrosis Exercise Working Group: Statement on Exercise Testing in Cystic Fibrosis Respiration 2015; 90:332-51.

2. Ruf K, Hebestreit H. Exercise-induced hypoxemia and cardiac arrhythmia in cystic fibrosis. J Cyst Fibros 2009; 8: 83–90.

3. Hebestreit H, Kieser S, Rudiger S, et al. Physical activity is independently related to aerobic capacity in cystic fibrosis. Eur Respir J 2006; 28:734–739.

4. Beaudoin N, Bouvet GF, Coriati A, et al. Combined exercise training improves glycemic control in adults with Cystic Fibrosis. Med Sci Sports Exerc 2017; 49:231–237

## QUALITY IMPROVEMENT

### A48 The Italian External Quality Assessment. Scheme for Sweat Test: the importance of a good communication with Laboratories

#### Marco Salvatore^1^, Giovanna Floridia^2^, Annalisa Amato^3^, Federica Censi^1^, Maria Chiara de Stefano^1^, Gianluca Ferrari^1^, Fabrizio Tosto^1^, Ettore Capoluongo^4^, Ubaldo Caruso^5^, Giuseppe Castaldo^6^, Carlo Corbetta^7^, Rita Padoan^8^, Valeria Raia^9^, Domenica Taruscio^1^ and Natalia Cirilli^10^

##### ^1^Centro Nazionale Malattie Rare, Istituto Superiore di Sanità, Roma, Italia; ^2^Unità di Bioetica, Istituto Superiore di Sanità, Roma, Italia; ^3^Lega Italiana Fibrosi Cistica, Roma, Italia; ^4^Laboratorio di Diagnosi Clinico Molecolare e Personalizzata, Fondazione Policlinico Universitario “A. Gemelli”, Roma, Italia; ^5^LABSIEM – Laboratorio per lo Studio degli Errori Congeniti del Metabolismo, Università degli Studi di Genova – DINOGMI, Genova, Italia; ^6^CEINGE – Biotecnologie Avanzate Scarl, Napoli, Italia & Dipartimento di Medicina Molecolare e Biotecnologia Medica, Università degli studi di Napoli Federico II, Napoli, Italia; ^7^SC Laboratorio di Riferimento Regionale per lo Screening Neonatale – Regione Lombardia, ASST Fatebenefratelli Sacco, Milano, Italia; ^8^Centro di Supporto Regionale Fibrosi Cistica, Dipartimento Pediatrico, Ospedale dei Bambini, ASST Spedali Civili, Brescia, Italia; ^9^Centro di Riferimento Regionale Fibrosi Cistica, Unità di Pediatria, Dipartimento di Scienze Mediche Traslazionali, Università degli Studi di Napoli, Federico II, Napoli, Italia; ^10^Centro di Riferimento Regionale Fibrosi Cistica, Dipartimento Materno Infantile, Ospedali Riuniti, Ancona, Italia

###### **Correspondence:** Marco Salvatore (marco.salvatore@iss.it)

**Background**

Sweat chloride is the gold standard test for the diagnosis of cystic fibrosis (CF). Internal and External quality controls are mandatory to assess precision and accuracy of test results and are recommended to improve the performance of the laboratory.

**Materials and methods**

In 2014 the National Centre for Rare Diseases (CNMR) of the Istituto Superiore di Sanità (ISS) established the Italian pilot external quality assessment scheme for CF sweat test (IEQA-ST). In 2015 this activity was recognized as a third party service carried out by the ISS. The program is performed once a year and participation is voluntary. Private and public laboratories, performing sweat test, are invited to participate. The IEQA-ST consists of a coordination board (managing the working plan) and of an evaluation board (assessing the performance of each laboratory) composed by experts from CNMR-ISS and Scientific Societies (SIFC, SIMMESN and SIBiOC). An IEQA-ST web-based utility is used to facilitate communication and data sharing among all parties. Each round consisted of the following items: 1) meeting between the boards to ameliorate items and criteria to be adopted in the upcoming round; 2) agreement by participants on the general rules and the assessment criteria; 3) delivery of 3 sweat like samples (including mock identification data, clinical and technical information; 4) collection of technical, institutional and analytical results (including report) from participants; 5) assessment of results; 6) a decisional meeting between the boards; 7) delivery of the final report to all labs; 8) annual meetings. The latest item represents the qualifying event of this plan. In 2016 the poor performance criteria was also adopted.

**Results**

The number of labs involved in this activity increased (from 10 to 16); analytical errors decreased by about 45% from 2016 ahead (2015: 11; 2016: 6; 2017: 6); clinical sensitivity (mainly wrong interpretation of the analytical result) errors increased (2015: 12; 2016: 9; 2017: 13); poor performance labs increased (2016: 3; 2017: 4). Main points of discussion during the annual meeting with all labs included: assessment criteria; final report; mock clinical information. Main feedbacks from labs: to maintain the annual meeting; to improve the content of the final report; to improve the communicating process.

**Conclusions**

The EQA-ST scheme is continuously evolving in accordance with national and international sweat test recommendations and feedbacks from participants. In this respect, laboratories opinion on the communication process, the technical support and the discussion about the assessment criteria are the driving force to improve the quality of the scheme.

**References**

1. Hebestreit H, Arets HG, Aurora P et al for the European Cystic Fibrosis Exercise Working Group: Statement on Exercise Testing in Cystic Fibrosis Respiration 2015; 90:332-51.

2. Ruf K, Hebestreit H. Exercise-induced hypoxemia and cardiac arrhythmia in cystic fibrosis. J Cyst Fibros 2009; 8: 83–90

3. Hebestreit H, Kieser S, Rudiger S, et al. Physical activity is independently related to aerobic capacity in cystic fibrosis. Eur Respir J 2006; 28:734–9.

4. Beaudoin N, Bouvet GF, Coriati A, et al. Combined exercise training improves glycemic control in adults with Cystic Fibrosis. Med Sci Sports Exerc 2017; 49:231–7

## EPIDEMIOLOGY AND NEWBORN SCREENING

### A49 Hypochloraemic Metabolic Alkalosis: insidious diagnosis

#### Mirella Collura^1^, Elisa.Parisi^2^, Annalisa Ferlisi^1^, Gabriella Traverso^1^, Lisa Termini^1^, Maria A Orlando^1^, Valeria Pavone ^2^, Oriana Bologna^1^, Ciro Corrado^3^, Caterina Di Girgenti^4^, Francesca Ficili^1^

##### ^1^U.O. Pediatria II per la FIBROSI CISTICA (CRR) e le Malattie Respiratorie. ARNAS Civico– Palermo, Palermo, Italy; ^**2**^Dipartimento di Scienze per la promozione della salute materno infantile G. D’Alessandro, Università degli studi di Palermo, Palermo, Italy; ^3^U.O. Pediatria ad indirizzo nefrologico ARNAS Civico, Palermo, Italy; ^4^U.O. U.O.D. Biologia Molecolare Arnas Civico, Palermo, Italy

###### **Correspondence:** Francesca Ficili (fficili@hotmail.com)

**Background**

Cystic fibrosis (CF) is the most common recessive genetic disorder in Caucasians. The suspicion of disease comes from the detection of typical symptoms, familiar history and newborn screening (NBS).

**Case reports**

We describe cases of two children who came to our observation for dehydration and metabolic alkalosis. NBS for Cystic Fibrosis resulted negative.

**Clinical Case 1**. Male 6 months. No genetic-metabolic diseases in family. History of recurrent ear infections. Some episodes of vomit, fever and hyporexia have been reported for some days. He appears in poor general conditions, moderate dehydration. EGA: PH: 7.52, HCO3: 36. Na: 129 mEq / l, K: 2.4 mEq / l, Cl: 91 mEq / l. ECG: hypokalemia. Hospitalized in our department he performs urinary electrolytes in the standard, renal function tests within normal limits. Eco-abdomen: negative, not signs of hypertrophic pyloric stenosis. He performs rehydrating therapy with physiological solution and Potassium Chloride with sudden improvement of the general clinical conditions and normalization of the ion and the parameters of the EGA within 24 hours.

**Clinical Case 2.** Male 10 months. No history of genetic diseases in family. Influenza episode at one month of life, bronchiolitis at the age of 3 months. From 15 days feverish, recurrent vomiting and hyporexia, reported weight loss (500 g). He comes to our observation for torpor and hyporeactivity. EGA: hypochloremic metabolic alkalosis. He performs urinary electrolytes, renal function tests within normal limits. Eco-abdomen: abdominal effusion, thickening of intestinal loops. Chest x-ray: multiple pulmonary densities. He performs rehydrating therapy with physiological solution and Potassium Chloride with sudden improvement of the general clinical conditions and normalization of the ion and the parameters of the EGA within 36 hours.

Both children performed screening for infective disease, renal tubular enzymes and Cortisol that were in the normal range. In stable conditions a sweat test was also performed and it resulted over the cut-off level of 60 mmol/l, therefore diagnosis of CF was performed, subsequently confirmed by genetics (2989+5G>A/4382delA; F508del/P5L, respectively).

**Conclusion**

NBS false negativity can be associated with laboratory errors, bad choice of cut-off values, pancreatic sufficiency, meconium ileus. Considering the high prevalence of false negativity, our centre is working to increase test sensitivity lowering the first IRT cut-off, to make a second test on the same sample (from 54 to 50 ng/ml) and defining the second IRT cut-off 40 ng/ml at 3-4 weeks of age.

In the detection of a hypochloremic metabolic alkalosis, in addition to CF, diseases such as RGE, APLV, metabolic diseases, adrenogenital syndrome, hypertrophic pyloric stenosis and tubulopaties must be sought.

Patients gave consent to data publication.

### A50 Cystic Fibrosis Screen Positive Inconclusive Diagnosis (CFSPID): Experience in Tuscany, Italy

#### Vito Terlizzi^1^, Gianfranco Mergni^1^, Roberto Buzzetti^2^, Claudia Centrone^3^, Lucia Zavataro^1^, Cesare Braggion^1^

##### ^1^Tuscany Regional Cystic Fibrosis Centre, Meyer University Children's Hospital, Florence, Italy; ^2^Epidemiologist, Bergamo, Italy; ^3^Diagnostic Genetics Unit, Careggi University Hospital, Florence, Italy

###### **Correspondence:** Vito Terlizzi (vito.terlizzi@meyer.it)

**Background**

The increased implementation of Cystic Fibrosis (CF) newborn screening (NBS) had led to identification of infants with a positive NBS test but inconclusive diagnostic testing, classified as “*CF screen positive, inconclusive diagnosis” (CFSPID)* in Europe [1-2]. We retrospectively evaluated the prevalence and clinical outcome of CFSPID patients by two NBS programs in the period 2011-2016 at CF Centre of Florence, Italy.

**Materials and methods**

We retrospectively evaluated CFSPID and CF patients diagnosed by CF NBS in the years 2011-2016 and followed until 31.12.2017. In this period a pilot project was aimed to assess the diagnostic impact of DNA analysis on the NBS algorithm (immunoreactive trypsin (IRT) - meconium lactase – IRT2). IRT was considered elevated for values above the 99^th^ centile laboratory cut-off. The CFSPID definition was according Munck A et al^1^. All CFSPID patients repeated SC ever 6 months and performed extended CFTR gene analysis (detection rate 98%). During follow up we reclassified children as: CF diagnosis, healthy carrier or healthy in presence respectively of at least two pathological SC or two normal SC for age and 1 or 0 CF-causing mutation. We kept the CFSPID definition when SC was persistently in borderline range or in presence of two CFTR mutations, at least one of which has varying clinical consequence (https://www.cftr2.org/).

**Results**

of 179684 babies screened from January 2011 to December 2016, 1520 (0.8%) screened IRT-positive value at day 3. Infants called to perform SC test were 359 by NBS algorithm and 181 by DNA analysis. We identified 32 FC diagnosis and 50 CFSPID (CF:CFSPID ratio 0.62:1). One of 179602 (0,0005%) cases resulted false negative by NBS and diagnosed by dehydration with hypochloremic metabolic alkalosis at 11 months. 20/50 (40%) CFSPID cases were diagnosed only by the IRT-DNA algorithm, 13/50 (26%) by only IRT-meconium lactase-IRT2, while both protocols identified the remaining 17 cases (34%). 36/50 CFSPID patients have a conclusive diagnosis on 31.12.2017: 5 (10%) FC, 16 (32%) healthy and 15 (30%) healthy carrier; 14/50 (28%) cases are asymptomatic with persistent borderline SC and followed as CFSPID (CF: CFSPID ratio 2,6:1). CFTR genetic analysis impacts on sensibility and positive predictive value (Table 1).

**Conclusions**

In six years CF: CFSPID ratio modified from 0.64:1 to 2.85:1 and 10% of CFSPID progressed to CF. Genetic analysis improved positive predictive value and identified a higher number of CFSPID infants progressing in CF.

**References**

1. Munck A, Mayell SJ, Winters V, Shawcross et al. ECFS Neonatal Screening Working Group. Cystic Fibrosis Screen Positive, Inconclusive Diagnosis (CFSPID): A new designation and management recommendations for infants with an inconclusive diagnosis following newborn screening. J Cyst Fibros. 2015; 14:706-713

2. Ren CL, Borowitz DS, Gonska T, et al. Cystic Fibrosis Transmembrane Conductance Regulator-Related Metabolic Syndrome and Cystic Fibrosis Screen Positive, Inconclusive Diagnosis. J Pediatr. 2017; 181S:S45-S51.e1.

**Table 1 (abstract A50).** Results

	CF vs (CFSPID+healthy)		(CF+CFSPID) vs healthy	
	IRT-lact-IRT2	IRT-DNA	IRT-lact-IRT2	IRT-DNA
Sensitivity %	87 (75-96)	90 (79-97)	78 (67-89)	90 (81-97)
Specificiy %	99.84 (99.83-99.86)	99.92* (99.91-99.93)	99.85 (99.83-99.87)	99.93* (99.92-99.94)
PPV^§^ %	11 (8-14)	19* (15-25)	13 (10-16)	26* (21-32)
NPV° %	99.997 (99.994-99.999)	99.998 (99.994-99.999)	99.994 (99.990-99.997)	99.997 (99.994-99.999)
Positive LR	555 (404-763)	1140* (829-1567)	514 (391-677)	1256* (955-1653)
Negative LR	0.13 (0.10-0.18)	0.11 (0.008-0.14)	0.22 (0.16-0.28)	0.10 (0.07-0.13)

^§^Positive predictive value; °Negative predictive value; LR= Likelihood ratios;*p<0.001

### A51 Cystic Fibrosis Screen Positive Inconclusive Diagnosis (CFSPID): nine years of experience in an Italian Cystic Fibrosis Centre (Naples)

#### Alice Castaldo^1^, Alida Casale^1^, Chiara Cimbalo^1^, Marco Sarno^1^, Clara Smaldone^1^, Valeria Raia^1^, Giuseppe Castaldo^2,3^, Antonella Tosco^1^

##### ^1^Cystic Fibrosis Center, Pediatric Unit, Department of Translational Medical Sciences University of Naples “Federico II”, Naples, Italy; ^2^Department of Molecular Medicine and Biotechnology, University of Naples Federico II, Naples, Italy; ^3^CEINGE- Advanced Biotechnology, Naples, Italy

###### **Correspondence:** Alice Castaldo (ali.castaldo@hotmail.com)

**Background**

Newborn screening (NBS) for Cystic Fibrosis (CF) identifies classic CF and most CFTR-Related Disorder (CFTR-RD), but also equivocal cases not fulfilling the diagnostic criteria for CF.

In 2015 the European CF Screening Neonatal Working Group defined such latter cases as “Cystic Fibrosis Screening Positive, Inconclusive Diagnosis” (CFSPID) according to the following criteria:

- Altered IRT at NBS
- Normal sweat chloride test (ST) (<30mmol/L) with 2 CFTR mutations, at least 1 of which with unclear phenotypic consequences or borderline ST (30-59 mmol/L) with none or 1 CFTR mutation
- Absence of symptoms

CFSPID identification is progressively increasing, and no defined guidelines are available for its follow-up.

**Materials and methods**

From January 2008 to December 2017, at Cystic Fibrosis Center of Naples (Pediatric Unit), 263.479 children were screened; 81 were identified as CFSPID (71(%, 44 males) and 33 as CF (29%, 17 males). These patients were monitored to define their evolution through clinical, biochemical, microbiological data and imaging procedure (median follow-up: 3.8 year. Range: 11 months-9.6 years).

**Results**

By NBS significant differences between CF and CFSPID were detected:

- I IRT: CF (mean ± DS: 140.1 ± 72.3 ng/dl) vs CFSPID (72.5 ± 21.4 ng/dl) p<0.001;
- II IRT: CF (147.7 ± 79.7 ng/dl) vs CFSPID (48.2 ± 10.7 ng/dl) p<0.001;
- ST: CF (77 ± 15.7 mmol/l) vs CFSPID (15.7 ±7.3 mmol/l) p<0.001;
- CF patients had 2 CFTR causing mutations, while among CSPID, 43 had 1 causing mutation and 1 with unclear phenotypic consequences and 38 had 2 mutations with unclear phenotypic consequences.

The follow-up of patients with CFSPID revealed that:

- these patients had less respiratory symptoms as compared to classic CF;
- none showed pancreatic insufficiency, meconium ileus nor metabolic alkalosis episodes;
- 8 cases developed a clinical feature suggestive of CFTR-RD (mean age: 4.5 years), one patient had a diagnosis of CF (Table 1.)

**Conclusions**

Our data suggest that:

1. based on our NBS program CFSPID is about 3 folds more frequent than classic CF (CFSPID:CF ratio 2.45:1) (the highest frequency reported so far);
2. few patients may evolve to CFTR-RD/CF;
3. the follow-up of three years suggested by the current literature is not sufficient to identify cases potentially evolving to CFTR-RD/CF;
4. no differences of IRT or ST were detected by NBS program in CF-SPID cohort that could predict evolution to CFTR-RD/CF.

**Reference**

1. Munck A, Mayell SJ, Winters V, Shawcross et al. ECFS Neonatal Screening Working Group. Cystic Fibrosis Screen Positive, Inconclusive Diagnosis (CFSPID): A new designation and management recommendations for infants with an inconclusive diagnosis following newborn screening. J Cyst Fibros. 2015; 14:706-713

**Table 1 (abstract A51).** Features of patients with diagnosis of CFTR-RD/CF

Patients	Characteristics	Diagnosis	Age of diagnosis	Sweat chloride	CFTR Genotype
1	bronchiectasis	CFTR-RD	8	28	F508del/Q1476X
2	bronchiectasis	CFTR-RD	8	27	1717-1G>A/5T12TG
3	recurrent pneumonia, nasal polyp	CFTR-RD	7	11	F508del/L997F
4	recurrent pneumonia, chronic sinusitis	CFTR-RD	3	35	R334Q/L997F
5	recurrent pneumonia	CFTR-RD	4	26	F508del/D1152H
6	recurrent pneumonia	CFTR-RD	3	12	S1426F/D1152H
7	recurrent pancreatitis	CFTR-RD	2	27	L732X/D1152H
8	recurrent pancreatitis	CFTR-RD	1	28	F508del/D1152H
9	sweat positive test	CF	3	62	c.2657+5G>A/5T-13TG

## PSYCOLOGY

### A52 Meaning of “time” after the lung transplantation

#### Paola Catastini^1^, Matteo Botti^2^, Cesare Braggion^1^

##### ^1^Cystic Fibrosis Regional Centre, Meyer Children’s Hospital, University of Florence, Florence, Italy; ^2^Santa Chiara Hospital, Department of Pediatric, University of Pisa, Pisa, Italy

###### **Correspondence:** Paola Catastini (catastini@iol.it)

**Background**

The lung transplant’s experience is a complex situation where a series of factors interact each others. A good physical condition in the post-transplantation period is not always a guarantee of positive emotional perception. The progressive resumption of life, sometimes are not experienced in a good emotional state, as if the patient could not take his “new time”. We report a clinical case of transplant cystic fibrosis patient where the psychological sessions showed this apparent contradiction.

**Materials and methods**

During the follow-up post-transplantation, we evaluated a patient, currently of 35 years old, with psychological assessment, for a period of six years (2012-2018). To assess depression and anxiety, at first evaluation in 2014, we administered respectively Hospital Anxiety Depression Scale (HADS) and State Trait Anxiety Inventory (STAI), and subsequently, as indicate by the Mental Health International Committee, we administered Patient Health Questionnaire (PHQ-9) and General Anxiety Disorders (GAD-7). Furthermore, during every evaluation, we administered Cystic Fibrosis Questionnaire-Revised (CFQ-R).

**Results**

During this period the general physical condition of the patient, the lung function and the nutritional state did not show critical aspects. The patient didn’t’ live any negative personal events. The score of depression, anxiety and quality of life after lung transplant are described in Table 1.

**Conclusions**

The results of psychological evaluation show higher levels of depression and lower score in QoL than the physical condition could be suggest. This case report underlines that a post-transplant, although without clinical complication, is not always an opportunity for the patients to re-discover all the aspects of the “new life”. This patient, like other similar in our experience, seems not to be able to enjoy his “time” of life. As well as, when the disease was disabling, they do not live their time even after transplantation. These patients are looking for a more intensive life and are worry about death. They have the perception of having lost “time” irremediably, due to cystic fibrosis. Knowing the average life expectancy in the post-transplant, they live like in a “turned hourglass”, where the transplant is the moment when the countdown started. We should ask ourselves if we are underestimating some deep aspects of their personal experience, consequently limiting the support we can give them. For this reason it is not only necessary do the psychological screening, but also, every time, the clinical interview. In this way we can really know the patient’s needs.

Patient gave the consent for publication.

**Table 1 (abstract A52).** Patient characteristics

	1 year before (2011)		2 years later (2014)	5 years later (2017)	6 years later (2018)
median FEV_1_	22,50%	transplant	67.10%	77.00%	79.80%
STAI1 S			61	/	/
STAI2 T			54	/	/
HADS			11		
PHQ-9			/	9 (mild)	15 (moderate-severe)
GAD-7			/	7 (mild)	3 (normal)
CFQ-R			70,1	77.6	70.4

## ENDOCRINE

### A53 Early Glucose Metabolism Alteration in Cystic Fibrosis Patients is associated with lower lung function

#### Silviana Timpano^2^, Chiara Perrotti^1^, Piercarlo Poli^2^, Martina Carne^3^, Claudia Conforti^1^, Elena Gennari^1^, Angela Andreoletti^1^, R. Padoan^2^

##### ^1^Graduate School in Paediatrics, Paediatric Clinic, ASST Spedali Civili Brescia, University of Brescia, Brescia, Italy; ^2^Regional Support Center for Cystic Fibrosis, Pediatric Department, ASST Spedali Civili of Brescia, Brescia, Italy; ^3^Graduate School in Dietetics, University of Brescia, ASST Spedali Civili of Brescia, Brescia, Italy

###### **Correspondence:** Piercarlo Poli (piercarlo.poli@gmail.com)

**Background**

Cystic fibrosis-related diabetes (CFRD) patients suffer from accelerated rates of pulmonary decline [1], also before the onset of overt CFRD [2-3]. This study aimed to determine if in a young CF population early alteration of glucose metabolism correlates with lung function or microbiological respiratory status.

**Materials and methods**

Clinical (BMI and cBMI), functional (FEV_1_ and FVC % predicted) and microbiological (presence of chronic Pseudomonas infection) data were collected from CF patients (>10yrs) who underwent a screening oral glucose tolerance test (OGTT) as part of their routine clinical care since September, 2016 to August, 2018. All data were collected at the time of the last OGTT test. Statistical analysis was performed using T-test to compare means, linear and multiple linear regression (Stata/SE 12.0) after normalization for age. A P-value <0.05 was considered significant in all tests.

**Results**

Screening OGTT was performed in 50 CF patients (24M/26F), mean age 17.01 years (± 4.63 yrs), median age 15.84 (range 10.60-31.55). CFRD was diagnosed in 9 (18%), in 11 (22%) an IGT resulted, while 60% (30 subjects) had a normal glucose metabolism. In the table (Table 1) clinical and metabolic data are presented.

**Conclusions**

Fasting blood glucose is not a helpful screening to diagnose early glucose metabolism alteration in CF patients. CF patients with an impaired glucose metabolism (IGT + CFRD) show a statistically significant lower FEV_1_ and FVC percent predicted. Chronic Pseudomonas colonization was higher in IGT and CFRD patients, however not statistical significant. Our data confirm the need of implementation of annual OGTT test in CF patients >10 yrs to identify early alteration in glucose metabolism, thus prompt nutritional advices and therapeutic measures might be implemented.

**Table 1 (abstract A53).** See text for description.

Characteristics	All (n=50)	NGT (n=30)	IGT (n=11)	CFRD (n=9)
Age (yrs)	17.01 ± 4.63	14.76 ± 2.47	19.41 ± 5.80	20.27 ± 2.86
BMI (kg/cm^2)^	20.05 ± 1.63	21.54 ± 2.09	20.10 ± 1.98	19.96 ± 1.64
cBMI	40 ± 26	40	30	50
Fasting blood glucose (mg/dl)	87.05 ± 8.0	85.73 ± 5.92	89.80 ± 13.09	85.33 ± 4.93
1-h OGTT blood glucose (mg/dl)	186.36 ± 55.25	170.33 ± 39.98	216 ± 68.21	215.67 ± 85.48
2-h OGTT blood glucose (mg/dl)	124.11 ± 40.19	104.8 ± 24.06	159.1 ± 15.08	215.33 ± 12.06
Fasting blood Insulin (μUI/ml)	7.68 ± 10.51	9.10 ± 12.08	4.7 ± 3.65	2
2-h OGTT blood Insulin (μUI/ml)	56.81 ± 41.04	48.69 ± 31.81	63.3 ± 44.86	74 ± 33.94
FEV_1_ (% p.)^a^	84.20 ± 21.59	90.4 ± 19.5	85.45 ± 21.33	65.88 ± 9.05
FVC (% p.)^b^	95 ± 17.20	100.77 ± 15.69	92.78 ± 18.20	80.5 ± 11.13
Chronic Pseudomonas infection^c^	46%	43.3%	54.5%	44.4%

^a^FEV_1_(%p): CFRD vs NGT p=0.0015; IGT vs NGT p=0.1; CFRD vs IGT p=0.035. CFRD + IGT vs NGT p= 0.0077

^b^FVC CFRD vs NGT p=0.0008; IGT vs NGT p=0.045; CFRD vs IGT p=0.1. CFRD + IGT vs NGT p= 0.0015

^c^Chronic Pseudomonas Infection _*X*_^*2*^*=*0.40, p>0.1 (NS)

**References**

1. Limoli DH, Yang, J, Khansaheb MK, Helfman et al. Staphylococcus aureus and Pseudomonas aeruginosa co-infection is associated with cystic fibrosis-related diabetes and poor clinical outcomes. European Journal of Clinical Microbiology & Infectious Diseases 2016; 35: 947-953.

2. O’Sullivan BP, Freedman SD. Cystic fibrosis. Lancet 2009; 373:1891–1904

3. Lek N, Acerini CL. Cystic fibrosis related diabetes mellitus-diagnostic and management challenges. Current diabetes reviews 2010; 6: 9-16.

### A54 Bartter syndrome or Pseudo-Bartter Syndrome in a Cystic Fibrosis infant? One face for two different diseases

#### Giuseppe F Parisi, Maria Papale, Lucia Tardino, Federico Mollica, Novella Rotolo, Salvatore Leonardi

##### Broncopneumologia Pediatrica e Fibrosi Cistica – Università degli Studi di Catania, Catania, Italy

###### **Correspondence:** Giuseppe F Parisi (giuseppeparisi88@hotmail.it)

**Background**

Cystic Fibrosis is a systemic pathology with associated loss of salts with sweat. Pseudo-Bartter syndrome (PBS) is an uncommon cause of metabolic alkalosis that has been described as a presenting feature of CF, as well as a complication in those with known disease. It is accompanied by chronic salt depletion and sometimes failure to thrive without severe dehydration. PBS is characterized by hyponatremic, hypochloremic metabolic alkalosis that mimics Bartter syndrome, but with no pathology in the renal tubules.

**Case report**

We present the case of a 1-year-old male with Eastern Europe origins, affected by CF in homozygous for the F508del. The patient comes to our attention very frequently due to repeated dehydration attacks during the whole year, despite the treatment with sodium chloride substitution (2-4 mmol/kg/day), recurrent vomiting and failure to thrive requiring 4 admissions to our ward without real improvement between hospitalizations.

**Conclusion**

The poor response to therapy and the extremely early onset of such manifestations have made us suspect that the patient may not have the CF-related PBS but the true Bartter syndrome.

Bartter is an autosomal recessive syndrome which manifests with an alteration of the salts reabsorption resulting in extracellular fluid volume reduction with possible low or normal blood pressure. It is caused by mutations of genes encoding proteins that transport ions across renal cells in the thick ascending limb of the nephron also called as the ascending loop of Henle. Specifically, mutations directly or indirectly involving the Na-K-Cl cotransporter are key. Loss of function of this reabsorption system results in decreased sodium, potassium, and chloride reabsorption in the thick ascending limb, as well as abolishment of the lumen-positive voltage, resulting in decreased calcium and magnesium reabsorption. Consequently, Patients with Bartter syndrome may also have elevated renin and aldosterone levels as compensation mechanism. There are 4-5 different type of Bartter syndrome, they are due to compound heterozygous or homozygous mutations in four genes that code for proteins involved in the reabsorption of chlorine in the ascending part of the loop of Henle.

Clinical features of patients are compatible not only with PBS but also with true Bartter syndrome due to poor response to therapy and failure to achieve well-being among critical episodes. For this reason, the genetic test for diagnostic confirmation was performed and we are waiting for the result.

Parents authorized data publication.

### A55 Cystic Fibrosis and alterations of the peripheral microcirculation

#### Maria Papale^1^, Giuseppe Fabio Parisi^1^, Carmela Gulizia^1^, Lucia Tardino^1^, Novella Rotolo^1^, Mario Ragusa^2^, Luigi Di Pino^2^, Salvatore Leonardi^1^

##### ^1^UOC Broncopneumologia Pediatrica e Fibrosi Cistica – Università degli Studi di Catania, Catania, Italy; ^2^Dipartimento Chirurgia generale e Specialità Medico chirurgiche – Università degli Studi di Catania, Catania, Italy

###### **Correspondence:** Maria Papale (mariellapap@yahoo.it)

**Background**

Cystic fibrosis (CF) is a multi-organ disease with various manifestations of various organs, but mainly concerning the pulmonary area. Also, pancreatic failure with consequent malnutrition, correlation with diabetes mellitus, obstructive azoospermia and the typical infertility in males, bone involvement with the appearance of osteoporosis have been widely demonstrated as cause of morbidity in CF. Furthermore, vascular complications have not been clearly discussed in clinical trials. The aim of this study is to demonstrate the involvement of the peripheral microcirculation in patients affected by CF, a little debated and in depth aspect in the literature.

**Materials and methods**

We analyzed 15 patients (age between 10 and 38 years, 6 males and 9 females). The patients underwent a capillaroscopic examination, a noninvasive and easily repeatable instrumental technique that in vivo and in real time study the morphological and functional characteristics of the microcirculation.

**Results**

The capillaroscopic examination showed morphological abnormalities of the peripheral microcirculation in 13 patients out of 15. 5 patients out of 13 have a reduced capillary density; 9 patients out of 13 have a tortuosity of the loops between 20 and 50%. Other highlighted alterations are t dilatation of the sub papillary plexus in 5 patients out of 13 and a moderate angiotectonic disorder in 10 patients out of 13 (Table 1).

**Conclusions**

The analysis carried out on the sample of patients examined, highlights a correlation between CF and t microcirculation alterations. This aspect, unlike other places of involvement, is not much discussed in the literature, but could underlines a close link with systemic microcirculation disorder. Therefore, we conclude that these alterations founded in CF may be linked with systemic disease and represent a cause of morbidity.

**Table 1 (abstract A55).** Results

Capillaroscopic changes	N (15)	%
Pathological capillaroscopy	13	86.6
Reduced capillary density	5	38.4
Tortuosity of the loops between 20-50%	9	69.2
Dilatation of the sub papillary plexus	5	38.4
Moderate angiotectonic disorder	10	76.9

### A56 Functional endoscopic sinus surgery and pathogens detection in cystic fibrosis airways

#### Elena Proietti, Grazia Dinnella, Giuseppe Gallo, Giulia Sannicolo, Matteo Giuliari, Manuela Pace, Cristina Guerzoni, Ugo Pradal

##### Centro di fibrosi cistica, unità di Pediatra, Ospedale di Rovereto APSS, Rovereto (TN) – Italy

###### **Correspondence:** Elena Proietti (elena.proietti@apss.tn.it)

**Background**

Airways colonization with multiresistant pathogens such as P. aeruginosa (PA), S. maltophilia (SM), S. marcescens (SEM) and Methicillin-Resistant Staphylococcus aureus (MRSA) in cystic fibrosis increases the frequency of exacerbations, accelerate lung function decline and affect prognosis. Treating such pathogens is a challenge in cystic fibrosis management and nasal polyposis may work as bacterial reservoir. The aim of our study was to investigate the effect of functional endoscopic sinus surgery (FESS) on pathogens detection in cystic fibrosis airways.

**Materials and methods**

In a retrospective observational study, we selected all subjects with diagnosis of cystic fibrosis with nasal polyposis who underwent a FESS in our center in Rovereto hospital. We cultured samples from lower airways (sputum) before and after surgery and samples from paranasal sinuses at surgery date. We compared: 1. the detection of multiresistant bacteria (PA, SM, SEM and MRSA) in lower airways and in paranasal sinuses. 2. the multiresistant pathogens in sputum before and after surgery.

**Results**

The study included 11 subjects. We detected PA in 8 subjects, SM in 4 subjects and SEM in 2 sunjects, no MRSA was detected. In the first analysis we observed different pathogens in lower airways and in sinuses samples in 8 out of 11 subjects: we detected SM (4 subjects) and SEM (2 subjects) in samples from lower airways only. In 2 subjects PA was found in lower airway only and in other 2 subjects in paranasal sinuses only. In the second analysis we observed that in 3 subjects we could not detect multiresistant pathogens before surgery, two of them became positive after surgery.8 subjects were positive before surgery, 4 of them became negative after FESS. The paired t test showed a decrease of 27% (95CI -71/+16 %, p-value 0.17) of probability in detection multiresistant bacteria after FESS.

**Conclusions**

We observed a discrepancy in bacterial detection using samples from lower airways and paranasal sinuses. Moreover, our study shows a negative trend in detection of multiresistant bacteria in sputum after surgery. This might suggest a positive effect of FESS in controlling pathogen colonization in cystic fibrosis.

